# Molecularly stratified hypothalamic astrocytes are cellular foci for obesity

**DOI:** 10.21203/rs.3.rs-3748581/v1

**Published:** 2024-02-09

**Authors:** Tibor Harkany, Evgenii Tretiakov, Luis Varela, Jasna Jarc, Patrick Rebernik, Sylvia Newbold, Erik Keimpema, Alexei Verkhratsky, Tamas Horvath, Roman Romanov

**Affiliations:** Center for Brain Research, Medical University of Vienna; Center for Brain Research, Medical University of Vienna; Yale University School of Medicine; Center for Brain Research, Medical University of Vienna; Medical University of Vienna; Center for Brain Research, Medical University of Vienna; Medical University of Vienna, Center for Brain Research; The University of Manchester; Yale University; Medical University of Vienna

## Abstract

Astrocytes safeguard the homeostasis of the central nervous system^1,2^. Despite their prominent morphological plasticity under conditions that challenge the brain’s adaptive capacity^3–5^, the classification of astrocytes, and relating their molecular make-up to spatially devolved neuronal operations that specify behavior or metabolism, remained mostly futile^6,7^. Although it seems unexpected in the era of single-cell biology, the lack of a major advance in stratifying astrocytes under physiological conditions rests on the incompatibility of ‘neurocentric’ algorithms that rely on stable developmental endpoints, lifelong transcriptional, neurotransmitter, and neuropeptide signatures for classification^6–8^ with the dynamic functional states, anatomic allocation, and allostatic plasticity of astrocytes^1^. Simplistically, therefore, astrocytes are still grouped as ‘resting’ vs. ‘reactive’, the latter referring to pathological states marked by various inducible genes^3,9,10^. Here, we introduced a machine learning-based feature recognition algorithm that benefits from the cumulative power of published single-cell RNA-seq data on astrocytes as a reference map to stepwise eliminate pleiotropic and inducible cellular features. For the healthy hypothalamus, this walk-back approach revealed gene regulatory networks (GRNs) that specified subsets of astrocytes, and could be used as landmarking tools for their anatomical assignment. The core molecular censuses retained by astrocyte subsets were sufficient to stratify them by allostatic competence, chiefly their signaling and metabolic interplay with neurons. Particularly, we found differentially expressed mitochondrial genes in insulin-sensing astrocytes and demonstrated their reciprocal signaling with neurons that work antagonistically within the food intake circuitry. As a proof-of-concept, we showed that disrupting *Mfn2* expression in astrocytes reduced their ability to support dynamic circuit reorganization, a time-locked feature of satiety in the hypothalamus, thus leading to obesity in mice. Overall, our results suggest that astrocytes in the healthy brain are fundamentally more heterogeneous than previously thought and topologically mirror the specificity of local neurocircuits.

## Introduction

Astrocytes, which belong to the broader class of astroglia, form a heterogeneous group of cells to maintain homeostasis within and provide defense for the central nervous system^1,2^. The term ‘astrocyte’ was introduced by Mihály Lenhossék in 1893^11^. During the past century, astrocytes were recognized for their unique cellular features^12^, particularly their plasticity, manifesting as morphological, biochemical, and functional remodeling, to allow for life-long adaptation^13^. Cardinal features include the dynamic encasement of synapses by astrocytic leaflets^14^, gap junction coupling for the synchronous operation of glial syncitia^15^, and physical attachment to the parenchymal basement membrane, thus creating the *glia limitans* to support the endothelial blood-brain barrier for nutrient transport into and metabolite clearance from the brain^1,16^. To do so, astrocytes benefit from their adaptive capacity to meet allostatic pressures within and beyond physiological boundaries^17–20^, including an ability to dedifferentiate, and to release both intercellular mediators^21^ (e.g., cytokines^22^, lipids^23,24^, and adenosine derivatives^25–27^) and nutrients through active transport^28^ in an inductive manner. Despite their features being fundamental for all of the brain’s physiological functions, the question of whether astrocytes are plastic and universal or instead possess cellular features specific to their location to support context-dependent changes in their transmitter-based communication with neurons to specify behaviors, warrants the exploration of molecular and spatial heterogeneity amongst these glial cells.

So far, electrophysiology^29^, optical imaging^29–32^, and single-cell sequencing methods^33^ were used to determine putative subtypes of astrocytes, and to match their state-dependent changes to brain regions and idiosyncrasies of neuronal activity. In doing so, the preferred classifiers used were originally designed for neurons^34–36^. These approaches lacked sufficient strength and resolution because of their inability to accommodate the dynamic selection and rapid ‘on/off’ rates of GRNs that are common to astrocytes^17,37^, which lack hardwired ‘terminal differentiation features’ and ‘lineage stability’ that typify neurons^38^. Accordingly, even if attempts were made to define astrocyte heterogeneity in the corticolimbic circuit, a laminar macrostructure with layer-specific transcriptional landmarks for neurons^31,33^, unique intralaminar features^39^ were gradually diluted and even lost when extrapolating those for astrocytes at large^6,7^. To overcome these fundamental shortcomings, cellular heterogeneity was replaced by functional state assignment, which is built on the assumption that astrocytes might express unique molecular features to reciprocate both metabolic requirements^6^, and the cellular competence of the neurons they communicate within a particular neurocircuit^32^. Nevertheless, the innate allostatic plasticity of astrocytes precluded the assignment of *bona fide* subregional identities. Thus, the persisting lack of an analysis pipeline specifically tailored to select astrocyte features for region-specific stratification remains a key hiatus in exploring cellular heterogeneity in the nervous system.

Here, we developed a walk-back feature-recognition method (‘AstroTRAP’) that uses iterative machine learning aimed to recognize the molecular signatures of spatially segregated astrocyte subpopulations under physiological conditions (that is, not including ‘reactive’ astrocytes associated with disease states). We benchmarked ‘AstroTRAP’ by integrating 16 open-label datasets on the hypothalamus^7,37,40–52^, a cytoarchitecturally complex brain region rich in neuronal subtypes of the many coexistent neuroendocrine command circuits, and thus challenging to analyze^8^. We developed a method that uses open reference sets of labeled data for the optimization of hyperparameters by measuring model performance metrics by iterative K-fold cross-validation, and then tested the model’s efficacy vs. a ‘ground truth’ (defined as astrocytes being uniform throughout). Thereafter, region-defining genes from whole hypothalamus reference datasets^7,43–45^ were extracted to process information on astrocytes from all subregions, including statistical comparisons. Thus, ‘AstroTRAP’ is procedurally distinct from ‘Hypomap’^6^, a comprehensive atlas of hypothalamic neurons created by unsupervised autoencoder-based representation. The use of semi-supervised machine learning thereby is superior to extract faint cellular signatures from transcriptional data, which are generally assigned as procedural ‘noise’ in algorithms presently available. Overall, we recognized astrocyte protogroups associated with food intake, lipid, sugar, and hormone metabolism. We have validated feature selection by neuroanatomy and functional interrogation of astrocyte-neuron interactions, particularly in the arcuate nucleus (ARC), and posit that astrocytes are critical to control bodyweight. Thus, we suggest that although hypothalamic astrocytes have ground-state signatures, they can be stratified under physiological conditions, including dynamic place- and use-dependent rearrangements, to sculpt neuroendocrine output.

## Results

### Data integration onto single-cell RNA-seq reference maps

**Figure 1:**
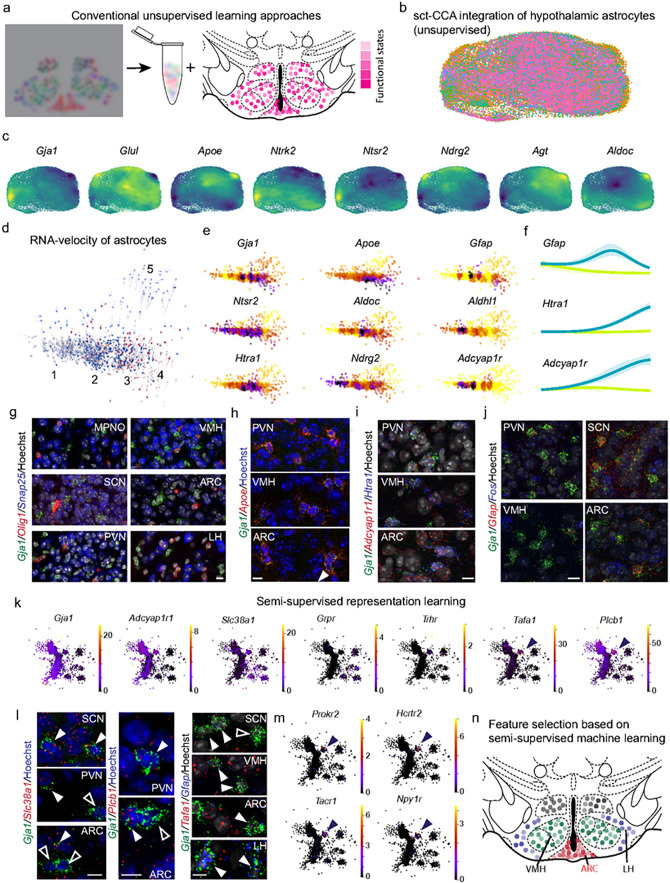
Cellular states of hypothalamic astrocytes revealed by single-cell RNA-seq. (**a**) Schema to suggest that unsupervised learning approaches, commonly used to analyze single-cell RNA-seq data, recognize functional states in astrocytes. (**b,c**) Single-cell-resolved rPCA-integration of data on hypothalamic astrocytes collated from 18 datasets^6,7,37,40–42,44–52,62,64,66^, including feature plots for select genes (**c**). (**d,e**) RNA velocity revealed a limited number of terminal states and the corresponding marker genes for hypothalamic astrocytes. (**f**) Reconstruction of pseudotime cellular trajectories for arcuate astrocytes by *CellRank*. Data from control and high-fat diet-fed conditions^37^ were shown. This approach distinguished subsets of cells with prominent gene expression changes (*blue*) vs. unchanged trajectories (*green*). Dimed colors label the 95% confidence interval. (**g-j**) *In situ* hybridization confirmed the expression of select markers in astrocytes (*Gja1* being a stable mark) in hypothalamic subregions. Other cell types were labeled by *Snap25* (neurons) and *Olig1* (oligodendrocytes) to show complementarity with *Gja1*. (**k**) UMAP plot, an outcome of semi-supervised representation learning, yielded higher group segregation with genes for receptors, signaling molecules, and ligands resolved in patterns amongst astrocytes yet without regional segregation. (**l**) *In situ* hybridization validated select markers from (**k**). *Solid arrows* point to *Gja1*^+^ astrocytes co-expressing the markers indicated. Conversely, *open arrows* denote the lack of co-existence. (**m**) Expression of genes encoding neuropeptide receptors in hypothalamic astrocytes. (**n**) Schema identifies the unresolved challenge of the spatial assignment of astrocytes that cannot succeed when using classical algorithms. *Scale bars* = 10 mm (**g-j,l**).

Despite numerous fundamental roles attributed to astrocytes^4,53,54^, their heterogeneity is generally overlooked in single-cell RNA-seq data because these cells do not conform to the classical, neuron-centric definition of ‘subclass identities’. This is because astrocytes do not adhere to stable developmental and functional ‘end-states’. Instead, they often exist in functionally distinct states^5,26,55,56^, molecularly characterized by a continuum of gene transcription events to produce graded responses^37,57^, thus accommodating environmental and metabolic conditions (Fig. 1a) in ever-changing physiological contexts. Any inducible property, therefore, hinders the resolution of (sub−)regional heterogeneity amongst astrocytes. If astrocytes had unique features that could assign them to specific brain areas and neuronal networks within, then integrating single-cell RNA-seq data from regional and subregional datasets (that is, select nuclei in a specific area) available to date could allow inferences to be made on astrocyte ‘subtypes’ and their positional segregation. Here, we have focused on the hypothalamus of adult mice (**Extended Data Table 1)**, which morphologically is an intertwined patchwork of nuclei^58–60^ and, at least at the level of neurons, contains functionally devolved neurocircuits. Accordingly, we sought to address whether astrocytes might be substantially different if recruited to support, interact with, or protect transcriptionally distinct neuronal subtypes^8,45,61–63^, with the expectation that astrocytes complement neuronal features for functional coupling.

To maximize spatial resolution, single-cell RNA-seq data on astrocytes were imported from all available datasets on the hypothalamus, and integrated from the paraventricular nucleus (PVN)^47^, arcuate nucleus/median eminence (ARC/ME)^37,40,62,64^, suprachiasmatic nucleus (SCN)^48,49^, ventromedial hypothalamus (VMH)^42,65^, ventroposterior hypothalamus (VPH)^50^, lateral hypothalamus (LH)^51,66^, preoptic area (POA)^52^, and median preoptic nucleus (MnPO)^46^, and mapped onto the four existing ‘whole hypothalamus’ datasets^7,43–45^ that served as reference matrices. An inherent problem of any single-cell method is potential cellular contamination, fundamentally biasing dimensional reduction, cell clustering, and other unsupervised machine learning methods. Astrocytes are particularly prone to contamination given their small nuclei and variable morphology. We mitigated any such bias by using *cell × gene* matrix filtering for quality control: *i*) sequencing reads were aligned to an optimized reference genome^46^; *ii*) *CellBender* was used to eliminate ambient RNA at differential detection rates^67^, while *Scrublet* removed doublets^68^;*iii*) mitochondrial and ribosomal mRNAs were manually scrutinized to limit discrepancies across batches, sequencing depth, and capture efficiencies. Lastly, we relied on the gene ontology platform^69^ to standardize classification, and applied intersectional criteria for manual annotation and filtering (**SI Methods, Extended Data Table 2**). Thus, we generated a dataset of 73,290 astrocytes, which lacked preferential selection for either sex or age (**Extended Data Table 1**).

### Unmasking astrocyte heterogeneity by unsupervised learning

Next, we have examined astrocyte heterogeneity by unsupervised learning algorithms, the least complex approach to analyze single-cell data^8,43,62^. We partitioned the consolidated dataset by a conventional Seurat-based pipeline (Fig. 1a) relying on reciprocal PCA integration based on Pearson residuals to ensure that batch effects did not artificially segregate the astrocytes (**Extended Data Fig. 1a**). Even if this approach embedded hypothalamic astrocytes (Fig. 1b), their differences were primarily defined by marker genes that reflected a metabolic ‘*ground state*’^10,37,70,71^ (that is, genes for amino acid synthesis (*Glul*), metabolism (*Apoe, S100b*), gap junction connectivity/maintenance (*Gja1*), and intercellular communication (*Slc1a2, Ndrg2, Ntsr2, Adcyap1r1*, and *Htra1*)), and were thus invariably central within the multidimensional cell matrix (Fig. 1c, **Extended Data Fig. 1b**). Alternatively, subsets of marker genes signified allostatic regulation (**Extended Data Table 3**) controlling the adaptation of astrocytes to environmental factors (e.g., genes for bioenergetics, amino acid transport, neurotransmitter utilization, and pH buffering), and distinguishing four non-overlapping astrocyte groups positioned at maximal distances from one another at the edge of the cell matrix: 1) *Gfap*-expressing(^+^) astroglia^3^, 2) high bioenergetic state defined by the rate of glucose utilization in and beyond the Szent-Györgyi-Krebs cycle (*Hadhb, Hacd2, Pla2g7, Ckb, Pygb, Tkt, Aldoc, Fth1*, and *Scd2*; **Extended Data Fig. 1b,c**), 3) high metabolic activity with a potential effect on nearby neurons (*Apoe, Hif1, Lcat, Lpcat3, Gli1*, and *Eno1*); and 4) excessive oxidative phosphorylation (*Cox1*, *Atp5f1a, Ndufs6*). These data corroborate metabolic differences amongst astrocytes in the hypothalamus through genes that drive activation states^70^ or metabolic competence^37^. However, conventional cell clustering failed to resolve *bona fide* stable astrocyte subtypes.

### Interrogating cell-state transitions by RNA velocity

Next, we deployed RNA velocity^57,72^ on a whole hypothalamus reference dataset^43^ to test whether cellular state transitions and transformations mask the intrinsic heterogeneity of astrocytes (Fig. 1d,e). RNA velocity mapped four ‘high-confidence’ and one ‘low-confidence’ terminal states that were generated from two initial *ground* states (Fig. 1d). Terminal states again differed in function-defining genes (e.g., *Aldh1l1, Ndrg2, Aldoc, Gfap; Lxn, Cst3, Slit2, Dagla, Mfn2, Daglb*; Fig. 1e and **Extended Data Fig. 1c**). Thus, both unsupervised and RNA velocity algorithms produced data reflective of the activity of astrocytes in each hypothalamic region rather than genuine subtypes. Noting that astrocytes can rapidly reorganize their physiological territorial domains and structural appearance under metabolic pressure (e.g. high-fat diet (HFD); Fig. 1f, **Extended Data Fig. 1d-f**)^37,40,65^ or stress (**Extended Data Fig. 1g,h**)^47^), leading to the redistribution of mutable astrocyte subgroups, neither method helped us to resolve spatially segregated stable astrocyte classes.

If the genes prioritized by the above algorithms were indeed insufficient to define astrocyte subtypes at particular hypothalamic locations, then their localization by *in situ* hybridization and/or histochemistry would reveal quasi-random cell distribution across the hypothalamus. Many of the ‘high-confidence’ markers (e.g., *Gja1, Apoe, Gfap, Adcyapr1r1, Fos, Lxn, Slit2*, and *Htra1*) uniformly labeled astrocytes, although at varying levels (Fig. 1g–j, **Extended Data Fig. 2**). Overall, our data suggest that classical bioinformatics used for terminally differentiated cells at stable end-states, including those successfully applied to hypothalamic neurons^43,44,62^, is unable to resolve the heterogeneity of astrocytes and assign those either to a hypothalamic subregion or a specific function through environmental predictors.

### Semi-supervised learning resolves functional segregation

Since an unsupervised algorithm did not unmask astrocyte heterogeneity, we introduced a semi-supervised learning procedure, which pairwise integrated (sub−)regional information (data on a specific region vs. whole hypothalamus reference dataset; **Extended Data Fig. 3a**) with an inflated number of anchor genes. To this end, we aligned a ‘whole hypothalamus’ dataset^43^ onto gene expression information from the ARC^37,40^, lateral hypothalamus (LH)^51^, MnPO^46^, POA^52^, PVN^47^, SCN^48,49^, VMH^41,42^, and VPH^50^ using the Harmony algorithm^73^ to simultaneously account for all technical (e.g., library versions, sequencing hardware, sequencing libraries, experimental batches) and biological (sex and age) factors (**Extended Data Table 1**). This approach did not yield regional clusters of astrocytes either, because functional states remained the predominant variables. Nevertheless, an unequal distribution of area-specific datasets on the cell continuum of hypothalamic astrocytes was observed (Fig. 1k), with some molecular features preserved in specific hypothalamic areas that unsupervised learning was unable to return.

Assuming there is indeed a molecular feature that is particularly well resolved by a semi-supervised approach, then *in situ* hybridization can be expected to support the uneven distribution of this specific mark. Amongst the suspect genes, *Grpr, Plcb1, Slc38a1, Tafa1* expression were prone to segregation (Fig. 1k,l). Particularly, a cluster of astrocytes was identified by their Plcb1 expression in conjunction with *Prokr2, Hcrtr2, Tacr1*, and *Npy1r*, suggesting cell-to-cell signalling by neuropeptides (Fig. 1m). These data were anatomically confirmed by the co-expression of *Gja1* with *Slc38a1, Tafa1, Plcb1*, and, although less frequent, with *Npy1r*, in some hypothalamic nuclei (Fig. 1l; **Extended Data Fig. 3b**). Thus, semi-supervised learning can topographically enrich gene sets with unequal expression that, however, mainly reflect activity-related differences in the hypothalamus.

### AstroTRAP: semi-supervised feature selection

To discriminate genes that define the (sub−)regional positions of astrocytes, and are independent of those signifying functional states, we modified semi-supervised sampling by progressively reducing the contribution of molecular features that reflect functional/metabolic states by extracting these as anchor genes through pairwise integration (Fig. 1n, **Extended Data Fig. 4**). Henceforth, shared features were identified based on their conserved variability across 48 paired integrations among four ‘whole hypothalamus’ datasets^7,43–45^, and twelve others, each individually sampling eight hypothalamic nuclei (ARC^37,40^, LH^51^, MnPO^46^, POA^52^, PVN^47^, SCN^48,49^, VMH^41,42^, and VPH^50^), and quantified as a function of rank aggregations across regions, and then across the four reference datasets (**Extended Data Table 3**). Thereby, we first resolved aggregated gene signatures non-specific to hypothalamic areas, with gene set enrichment consistently identifying (that is, regardless of the stringency of ambient RNA removal with either 0.001 or 0.01 FDR, or even without correction) ‘regulation of cellular metabolic process’ (GO:0031323, *p* = 4.18 × 10^−7^), ‘*organonitrogen compound metabolic process*’ (GO:1901564, *p* = 7.28 × 10^−7^), ‘*regulation of primary metabolic process*’ (GO:0080090, *p* = 4.09 × 10^−6^), ‘*regulation of signal transduction*’ (GO:0009966, *p* = 4.73 × 10^−5^), and ‘*regulation of response to stimulus*’ (GO:0048583, *p* = 1.25 × 10^−4^; **Extended Data Table 3**). Analysis of the same features with the *ensemble* method^74^, selected for discrimination among the eight hypothalamic subregions, resulted in the enrichment of genes for development. Thus, in three independent prefiltering methods, we consistently obtained the following ‘*top 5*’ gene categories: ‘*nervous system development*’ (GO:0007399), ‘*system development*’ (GO:0048731), ‘*anatomical structure development*’ (GO:0048856), ‘*multicellular organism development*’ (GO:0007275), and ‘*developmental process* (GO:0032502), when using c^2^ statistics^75^, ANOVA *F*-value^76^, or mutual information score^77^ selection methods. Alternative refinement methods, be these *XGBoost* and L1-regularised logistic regression (*logit*), resulted in similar ‘*top 5*’ results (for XGBoost: ‘*nervous system development*’ (GO:0007399, *p* = 8.38 × 10^−19^), ‘*system development*’ (GO:0048731, *p* = 6.73 × 10^−18^), ‘*neurogenesis*’ (GO:0022008, *p* = 1.21 × 10^−14^), ‘*regulation of biological quality*’ (GO:0065008, *p* = 1.56 × 10^−14^), and ‘*multicellular organism development*’ (GO:0007275, *p* = 2.29 × 10^−14^), and for logit: ‘*system development*’ (GO:0048731, *p* = 4.62 × 10^−29^), ‘*multicellular organism development*’ (GO:0007275, *p* = 4.20 × 10^−27^), ‘*anatomical structure morphogenesis*’ (GO:0009653, *p* = 2.95 × 10^−22^), ‘*nervous system development*’ (GO:0007399, *p* = 1.07 × 10^−^21), and ‘*animal organ development* (GO:0048513, *p* = 2.65 × 10^−21^; **Extended Data Table 4**). Overall, these findings revealed that patterning *via* morphogenic transcription factors (TFs) are irrelevant to the functional/metabolic states of astrocytes.

**Figure 2:**
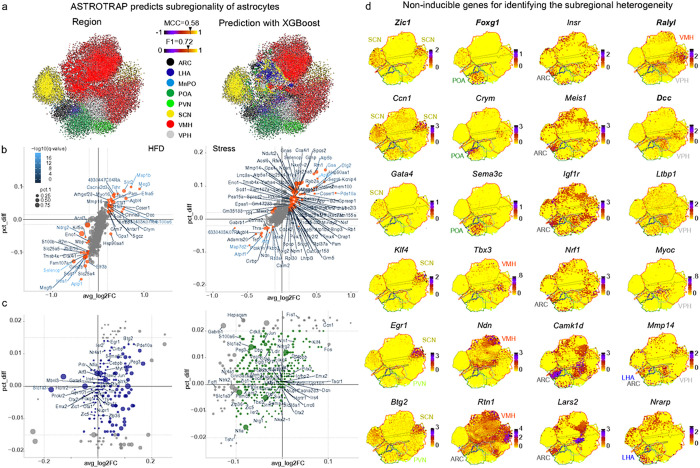
Implementation of ‘AstroTRAP’ for the spatial assignment of hypothalamic astrocytes. (**a**) Supervised UMAPs for integrated nucleus-specific hypothalamic datasets (ARC^37,40^, LH^51^, MnPO^46^, POA^52^, PVN^47^, SCN^48,49^, VMH^41,42^, and VPH^50^) color-coded with their true labels (*left*), and as predicted by AstroTRAP (*right*). *Top*: Optimized outcome when using an *XGBoost*-based model with the highest reliability scores (MCC, F1). (**b,c**) Altered gene expression in astrocytes from the ARC (*left*)^37^ and PVN^47^ (*right*) upon exposure to high-fat diet (HFD) and chronic social defeat stress, respectively. Genes that had undergone significant changes (that is, inducible) were shown in (**b**). Unchanged gene sets (considered non-inducible) were marked in (**c**). (**d**) Hexagonal bin (hexbin) plots depicting genetic marks specific to hypothalamic subregions identified by AstroTRAP. UMAP space was partitioned by using the color codes from (**a**).

Next, the efficiency of regional resolution was measured by searching across a hyperparameter grid and identifying best estimators, thus cross-validating the model’s performance metrics: weighted *F*-measure, as the harmonic mean of precision and recall, and *Mathews correlation coefficient* (MCC, from −1 to 1), improved the classification of unbalanced samples, a problem inherent to samples for specific hypothalamic nuclei and 10x Genomics libraries. Upon this optimization, we could construct two support vector machine (SVM)-based classifiers, which succeeded in predicting the regional origin for each astrocyte from any of the scRNA-seq datasets. Accordingly, our workflow selected 1,406 genes by *logit*-based feature selection with L1-regularization, and achieved an MCC of 0.441, and weighted *F*-measure of 0.627 on the distinct evaluation set (**Extended Data Fig. 5a** and **Extended Data Tables 4,5**). Alternatively, *XGBoost*-based feature selection collated 211 genes and a robust SVM classifier, which was superior to the logit-based classifier with an MCC of 0.578 and a weighted *F*-measure of 0.723 on the distinct evaluation (Fig. 2a, **Extended Data Fig. 5b**, and **Extended Data Tables 4,5**). Thus, we constructed an algorithm, termed ‘AstroTRAP’, that could assign astrocytes from an unspecified mixture of cells to hypothalamic areas with high fidelity.

### Stable gene sets, including neuropeptide receptors, define hypothalamic astrocytes

Even though a novel algorithm might perform correctly on a dataset representing physiological conditions, it could become biased or fail with the added complexity of an experimental (or environmental) challenge, because of the many inducible genes that exist in astrocytes. To address the question of gene induction as a potential confound, we tested differentially-expressed genes in open-source scRNA-seq data on diet-induced obesity^37^ and chronic stress due to social defeat^47^ (Fig. 2b). These paradigms induced expression shifts in genes for ‘intercellular signaling’ (e.g., *Slit2, App, Cacna2d3, Dcc, Efna5, Efr3b, Gabrb3, Gnas, Grin2b, Grm5*, *Grm7, Pde10a, Tshr*) and ‘allostasis’ (e.g., *Atp5b, Cirbp, Eno1, Fos*, *Gaa, Hsp90aa1, Ndufc2, Selenop, Slc25a4, Slc25a5*). When particularly subsetting stable genes (that is, non-inducible upon manipulation), we retrieved a largely unchanged gene set that also anchored AstroTRAP under physiological conditions (Fig. 2c, **Extended Data Table 4**), and which remained sufficient to define regional identity (Fig. 2d). Thus, genes specifying astrocytes in six of eight hypothalamic areas are: Insr, *Meis1, Igf1r, Nrf1, Camk1d, Lars2* for ARC, *Nrarp* for LH, *Zic1, Ccn1, Gata4, Klf4* for SCN, *Foxg1, Crym, Sema3c* for the POA, *Tbx3, Ndn* for VMH, and *Dcc, Ltbp1, Myoc* for VPH. Moreover, striking subregional genes were in *Rtn1* for ARC and VMH, *Mmp14* for ARC, VPH and LH, *Egr1*, *Btg2* for SCN and PVN, *Cirbp* for MnPO, for POA and SCN, and Ralyl for VMH and VPH (**Extended Data Table 6**).

**Figure 3:**
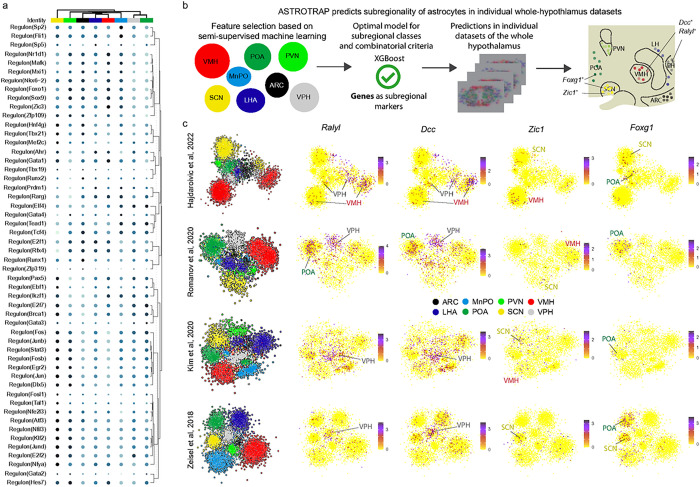
Implementation of ‘AstroTRAP’ for the regional assignment of genes and gene regulatory networks (GRNs). (**a**) Spatial assignment of astrocytes extracted from ^44^ to hypothalamic areas (color-coded as in (**b**)), as well as the analysis of GRNs using the SCENIC workflow. Transcription factors are highlighted as specific marks of regionality. (**b**) Schema of how to extract spatial information from unsorted datasets using AstroTRAP. (**c**) AstroTRAP-derived regional enrichment in hypothalamus datasets that lacked spatial information. Feature plots for *n* = 4 selected genes are shown. Based on data from 4 ‘whole’ hypothalamus datasets^7,43–45^, a consensus existed on their spatial assignments, which was also consistent with their gene expression data in the integrated dataset used to benchmark spatial information (see: Fig. 2d).

When interrogating the determination of function, AstroTRAP assigned many genes encoding neuropeptide (and hormone) receptors (e.g., *Trhr*(**Extended Data Fig. 6a,b**),*Prokr2* (**Extended Data Fig. 6a,c**), *Hcrtr2*(**Extended Data Fig. 6a,d**), *Tacr1* (**Extended Data Fig. 6a,e,f**), *Grpr* (**Extended Data Fig. 6a,g, Extended Data Fig. 7**)), receptor subunits for fast and slow neurotransmission (**Extended Data Fig. 8**), G proteins for signal transduction, and intracellular receptors (**Extended Data Fig. 8, Extended Data Table 4,6**) at high precision for astrocytes population in specific hypothalamic areas. The expression of *Npy* receptors was particularly significant in astrocytes adjoining neurons of the core feeding circuit, and regulated by upstream ‘master’ TFs, such as *Jun* and *Bclaf1* for *Npy1r*, and *Rfx3* for *Npy5r* (Fig. 3c, **Extended Data Fig. 5**). These data suggest functional co-specification between astrocytes and neurons to produce neuroendocrine output by co-opting.

### Transcription factors and their GRN in astrocytes

Besides function-relevant genes, the very core genes that defined regional identity were TFs (prominently *Cdk8, Foxg1, Mytl1, Nr4a1, Otp, Otx2, Peg3, Six6, Tbx3*), critical for patterning^78^ and the activation of distinct differentiation programs^44,45^ (Fig. 2d,3b, **Extended Data Fig. 5b,c; Extended Data Fig. 8**). Particularly, *Foxg1* (**Extended Data Fig. 9a**) specified the POA, *Nr4a1*(**ED Fig. 6c**), *Klf4, Lrrc6, Zfp36, Zic1, Gata4* could serve as markers for SCN (or more broadly, anterior hypothalamus); *Six6, Peg3, Cdk8, Myt1l, Tbx3, Nkx2–1, Isl1* marked astrocytes in tuberal subregions (ARC and VMH; **Extended Data Fig. 9b**; including *Otp, Nr5a1, Dlx1* specific to VMH), and *Emx2, Otx1, Otx2, Pitx2* (**ED Fig. 9c**) defined the posterior hypothalamus (VPH; Fig. 2d and 3c, **Extended Data Fig. 5c**, **6** and **9**). These data suggest a significant overlap exists between the TF signatures of astrocytes and neurons in specific hypothalamic nuclei (for neurons see Ref.^44^), suggesting shared developmental origins.

Next, we expanded our analysis to GRNs to evaluate region-specific developmental programs driven by distinct TFs and signal-receptor tuning, alike reported earlier for developing neurons in the hypothalamus^79^. Several GRNs used by neurons were also expressed by astrocytes (e.g., *Dlx1, Foxg1, Isl1, Nkx2–1/2–2, Nr3c1, Nr5a1, Otp, Six3, Tbx2/3/21*), yet at lower levels^44^ (Fig. 3a), thus placing these TFs in a novel non-neuronal cellular context.

### Benchmarking AstroTRAP on unsorted datasets

To estimate the correctness of nucleus assignment in further analyses, including GRN assessment (Fig. 3a) in datasets without subregional labels of astrocytes^7,43–45^, we tested AstroTRAP on four datasets of the whole hypothalamus that lacked spatial information (Fig. 3b,c, **Extended Data Fig. 9a, 10a-c, Extended Data Table 6**). Therefore, we sorted data from these scRNA-seq datasets^7,43–45^ against a ‘training dataset’ containing regional data from the eight hypothalamic nuclei resolved earlier^7,37,40–52^, and achieved accurate spatial assignment using test markers like *Ralyl*, *Dcc, Zic1*, and *Foxg1* (Fig. 3b,c, **Extended Data Fig. 9a**).

However, regionally restricted (that is, existing in small cell fractions) and lowly-expressed TFs were difficult to detect in datasets over the ‘whole’ hypothalamus (‘dilution effect’). For instance, the orthopedia homeobox gene (*Otp*), marking a rare subtype of astrocytes, was barely detected in unsorted astrocytes (**Extended Data Fig. 10a,b**). Yet, the use of area-specific training datasets ensured minimal loss of information when processing bulk data. Moreover, combining AstroTRAP with *in situ* hybridization could anatomically validate the existence of even rare *Otp*^+^/*Gja*^+^ astrocytes in, e.g., the ARC, LH, and VMH (**Extended Data Fig. 10c**). Overall, AstroTRAP could effectively assign astrocytes to specific hypothalamic areas, and allow for hypotheses be made on astrocyte functions given the regional enrichment of regulons, receptors, and signaling molecules.

### AstroTRAP preserves inducible marks

**Figure 4:**
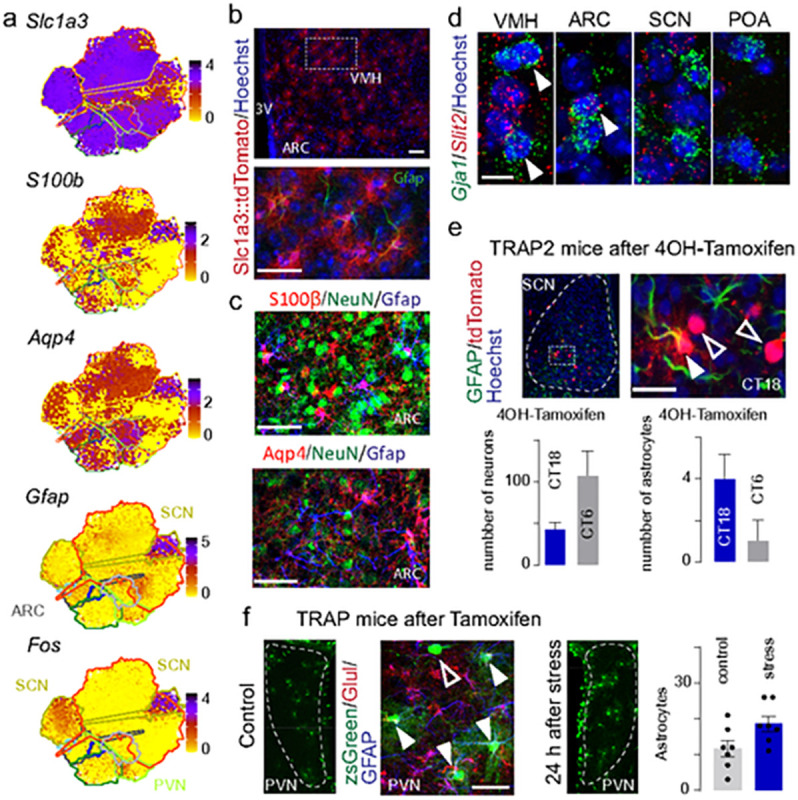
Benchmarking canonical glial markers and inducible genes in the hypothalamus. (**a**) Hexagonal bin plots for *Slc1a3, S100b*, *Aqp4, Gfap, and Fos*, superimposed on a supervised UMAP, shows area-specific gene enrichment. UMAP space was partitioned as in Fig. 2a. *Gfap* and *Fos*, being inducible genes, had unequal distribution across hypothalamic areas. (**b**) Lineage tracing of *Slc1a3*^+^ cells in the postnatal hypothalamus, with tamoxifen injected on P21. Although only ARC and VMH were shown, labeled cells morphologically resembling astrocytes were indiscriminately found in the hypothalamic nuclei. (**c**) S100b, AQP4, GGFAP (astrocyte markers), and NeuN were localized in ARC. (**d**) *In situ* hybridization for *Slit2*, another inducible gene in astrocytes, showed indiscriminate distribution of *Slit2*^+^/*Gja1*^+^ cells in hypothalamic areas under control conditions. (**e**) In TRAP2 mice induced with 4OH-tamoxifen, cFOS^+^/GFAP^+^ astrocytes concentrated in the SCN, and showed diurnal fluctuations. *Arrows* point to star-shaped protoplasmic astrocytes. Quantification is from *n* = 3 male mice per condition. (**f**) TRAP mice induced with tamoxifen and exposed to acute stress harbored significantly more *Fos*^+^ astrocytes (*solid arrows*) that co-expressed glutamate-ammonia ligase (GLUL) and GFP in the PVN vs. controls. *Open arrows* indicate neuron-like cells. Quantification is from *n* = 7 male mice/condition, *p* = 0.017 of two-tailed *t*-test with Welch’s correction. The PVN was demarcated by its higher cell density than surrounding areas. *Scale bars* = 50 mm (**b,c**), 40 mm (**e,f**), 10 mm (**d**).

Our focus on building an analysis tool that produces spatial information by segmenting inducible and non-inducible features might be prone to losing information on inducible marks. This could limit the power of analysis in experimental contexts. Therefore, we determined if the representation of functional states in astrocytes is changed by focusing on the most common prototypic cellular marks for astrocytes: regional enrichment in (or lack thereof) *S100b, Aqp4, Gfap, Slc1a3* in AstroTRAP (Fig. 4a) were compatible with those seen by reporter gene induction *in vivo* and histochemistry (Fig. 4b,c)^1,13,80^. Similarly, AstroTRAP mapped *Fos* (and *Slit2*), a prototypic inducible gene that undergoes ‘on/off’ cycles^49,70^ in a circadian fashion in the central pacemaker circuit of the SCN (being ‘on’ during the dark phase), at unequal distribution (Fig. 4a and Fig. 2b,c). Likewise, AstroTRAP predicted *Fos*^+^ astrocytes to populate the PVN upon stress (Fig. 4f). These data were confirmed for both nuclei by both cell-resolved anatomy and *Fos*^TRAP^ mice (Fig. 4d–f). These results suggest that inducible genes could appear as differentially expressed genes in AtroTRAP, yet excluded from defining regional identity.

### Regional differences in astrocyte-neuron communication

**Figure 5:**
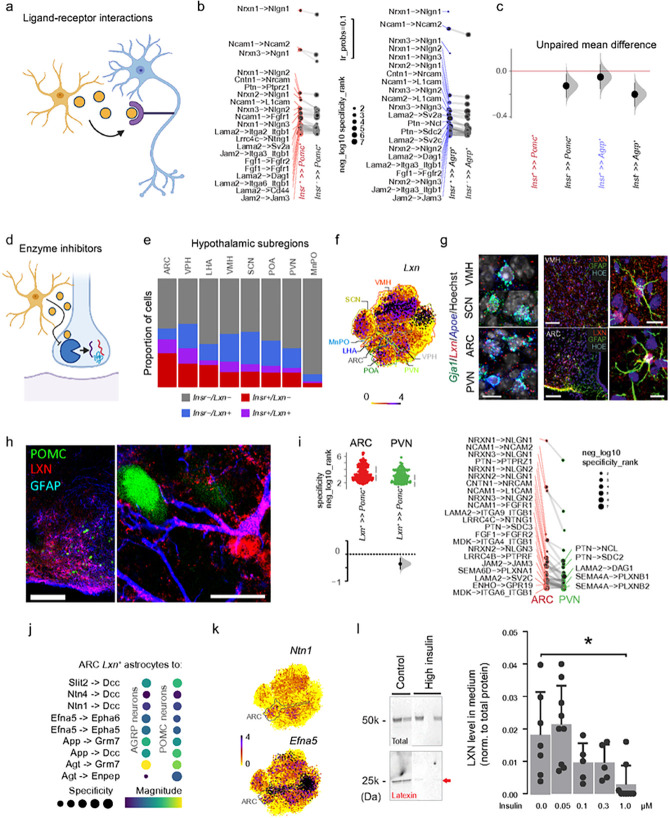
Modes of communication between astrocytes and Agrp^+^ or Pomc^+^ neurons in ARC. (**a**) Illustration of possible ligand-receptor interactions between astrocytes and neurons. (**b,c**) *CellChat* applied to single-cell RNA-seq data reconstructed ligand-receptor interactions between subgroups of astrocytes, subset by being either *Insr*^*+*^ or *Insr*^−^, and *Pomc*^+^ vs. *Agrp*^+^ neurons. Interactions exceeding ± 15% were indicated. (**d**) Shema of possible metabolic interactions between astrocytes and neurons driven by differential enzyme expression. (**e**) Relative densities of *Ins*^+^, *Lxn*^+^ and *Ins*^+^/*Lxn*^+^ astrocytes (normalized) in *n* = 8 hypothalamic areas, based on single-cell RNA-seq data (ARC^37,40^, LH^51^, MnPO^46^, POA^52^, PVN^47^, SCN^48,49^, VMH^41,42^, and VPH^50^). (**f**) Feature plot showing the expression of *Lxn* in astrocytes from all areas computed in (e). (**g**) *Left*: *In situ* hybridization of *Lxn* in VMH, ARC, PVN, SCN. *Gja1* and *Apoe* marked astrocytes. *Right*: Immunohistochemistry for LXN and GFAP in VMH and ARC. (**h**) The end-feet of LXN^+^/GFAP^+^ astrocytes preferentially enwrapped POMC^+^ neurons. (**i**) Computational comparison of potential interactions between *Lxn*^+^ astrocytes (residing in ARC or PVN) and POMC^+^ neurons. Left: Calculation by Liana. Right: Data from *CellChat*. Data were filtered for signaling pathways with ligands specific to *Lxn*^+^ astrocytes (all those also found in PVN were excluded) and receptors in POMC^+^ neurons. Local astrocytes in ARC had many more matches than those from PVN. Pathways that were specific to the ARC were labeled to the right. (**j**) Signaling interactions reconstructed with *Liana* between *Lxn*^+^ astrocytes and either *Pomc*^+^ or Agrp^+^ neurons in ARC. Sizes of the labels reflect the level of ‘specificity’ across the groups labeled. (**k**) Feature plots for *Ntn1* and *Efna5* for astrocytes in ARC. (**l**) Extracellular LXN released from astrocytes under control conditions and when stimulated by insulin *in vitro. Scale bars* = 100 mm (**g,h**), 10 mm (*high power micrographs*).

To test predictions on astrocyte-neuron communication, we focused on a well-defined neurocircuit of the ARC, for which both fast neurotransmitter and neuropeptide codes have been resolved previously ^62,81–85^. Firstly, we have estimated the array of interaction pairs between ligands expressed by astrocytes and their cognate receptors in neurons (Fig. 5a). Notably, *Insr* (encoding the insulin receptor) was found as an ARC-specific spatial marker for astrocytes (Fig. 2d). To focus on the putative role of *Insr*, we computationally assessed ligand-receptor coupling between *Insr*^*+*^ vs. *Insr*^−^ astrocytes with either proopiomelanocortin (*Pomc*^+^) or agouti-related peptide/neuropeptide Y (*Agrp*^+^)-expressing neurons. To this end, we established both ligand-receptor pairing probability (*lr_probs*) by *CellChat*^86^ with a manually curated shortlist of ligand-receptor pairs (Fig. 5b), as well as *Liana-py*^87^ that aggregates probabilistic estimates from most well-established methods into a single metric (*neg_log10_specificity_rank*) based on the same or expanded databases (Fig. 5b,c; see alsodotplots in **Extended Data Fig. 12,13**).

We observed the preferential coupling of *Insr*^+^ astrocytes with both *Pomc*^+^ and *Agrp*^+^ neurons across methods and databases, which was indicated by the higher number of ligand-receptor pairs (vs. *Insr*^−^ astrocytes; Fig. 5b), and by overall enrichment in signal flow from *Insr*^+^ astrocytes to both *Pomc*^+^ and *Agrp*^+^ neurons (Fig. 5c) based on *bootstrap*-coupled estimation^88^. Particularly high probability of difference existed for neurexin-neuroligin, neural cell adhesion molecule (*Ncam1*)-*Fgfr1,Ncam2/L1cam*, contactin (*Cntn1/Nrcam, Nfasc/Cntn1, Cntn2/L1cam*), pleiotrophin (*Ptn-Alk/Ncl/Ptprz1/Sdc1/2/3/4*) signaling (Fig. 5b) for *Insr*^+^ vs. *Insr*^−^ astrocytes.

Subsequently, we explored the possibility to incorporate cell-cell signaling routes predicted for the ARC by AtroTRAP, but not included in ligand-receptor databases (e.g., enzymes with a putative action extracellularly; Fig. 5d). Particularly, we analyzed possible standalone functions for *Insr*^+^ astrocytes that could be placed downstream to insulin signaling, noting that insulin itself is one of the most powerful exogenous stimuli for neurons of the ARC^89,90^. Among the genes (Fig. 5e), we have identified latexin (*Lxn*), whose cystatin-fold structure makes it ideal to inhibit zinc-dependent metallocarboxypeptidases (MCPs)^91–93^. When (and if) released from astrocytes, *Lxn* could, hypothetically, inhibit MCPs that cleave preproneuropeptides/preprohormones (e.g., *Pomc*), allowing for the specific tuning of the ARC neurocircuit (Fig 5d). When mapping regional specificity for *Lxn* expression across eight datasets, we found that *Lxn*^+^ astrocytes constitute the largest fraction in ARC, with many of them co-expressing *Insr* (c^2^Pearson (1) = 14.48, p = 1.42 × 10^−4^; Fig. 5e, **Extended Data Fig. 11**). Conversely, when we neglected the interaction between I*nsr* and *Lxn*, the ARC was the only hypothalamic region where *Insr* became insignificant (c^2^gof (1) = 2.66, *p* = 0.10). These data suggest functional coupling between the insulin receptor and *Lxn* expression. The tight relationship between *Insr* and *Lxn* in the ARC is likely physiologically important because, in contrast to *Insr, Lxn* is not regionally restricted in the hypothalamus (Fig. 5f, as also shown by both *in situ* hybridization and histochemistry (Fig. 5g). High-resolution microscopy further demonstrated that *Lxn*^+^ astrocytes frequently enwrap POMC^+^ neurons (vs. AgRP^+^ neurons; Fig. 5h), suggesting that latexin, when secreted, could affect the processing of POMC, which is a multifunctional neuropeptide^81,93^.

To support the preferential coupling between *Lxn*^+^ astrocytes and *Pomc*^+^ neurons in the ARC, we have compared *Lxn*^+^ astrocytes in the ARC with *Lxn*^+^ astrocytes in the PVN, and *Lxn*^−^ astrocytes in the ARC with *Pomc*^+^ and *Agrp*^+^ neurons: differences existed in both spatial resolution (Fig. 5i) and HFD-dependence (**Extended Data Fig. 12,13**). We identified Netrin-deleted in colorectal cancer (*Dcc*) and *Efna5-Epha5/6* signaling amongst pathways that differed (Fig. 5j,k). Again, the ligands (*Ntn1* and *Efna5*) were broadly expressed in astrocytes, whereas the receptor (*Dcc*) was prominent only in *Pomc*^+^/*Anxa2*^+^ neurons^62^.

For LXN to modulate MCPs, either extracellularly or after import/uptake into *Pomc*^+^ neurons, it shall be released in an insulin-dependent fashion. Therefore, we measured LXN levels in extracellular media (supernatants) of primary astrocytes isolated from the ARC in response to ascending concentrations of insulin. LXN was recovered from cell supernatants, too. Insulin inhibited LXN release from astrocytes (Fig. 5l), which instead accumulated intracellularly (**Extended Data Fig. 14**). These data suggest that LXN might function as a gliotransmitter affecting a specific neuronal contingent in the ARC.

### Astrocytes modulate food intake

**Figure 6:**
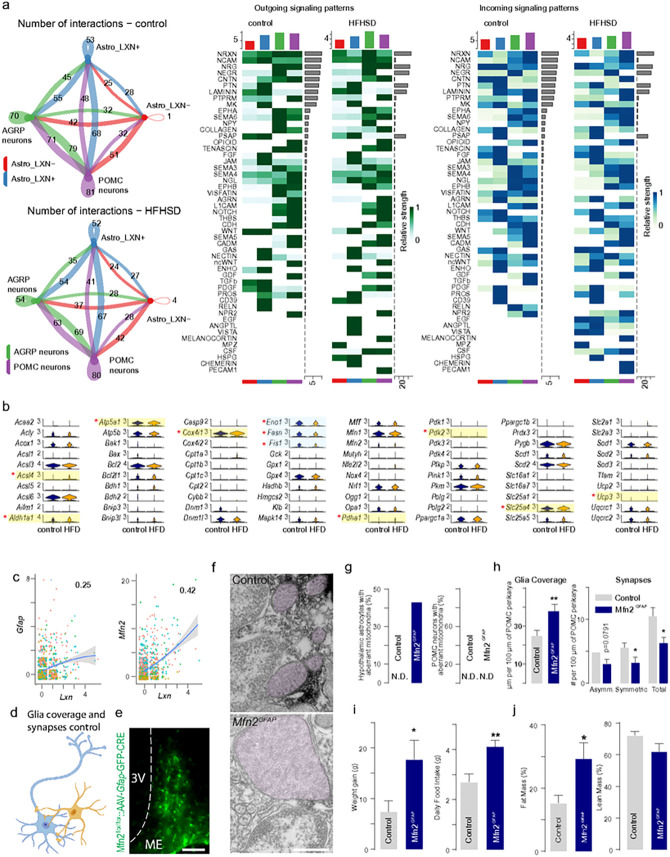
*Mfn2* expression and function in astrocytes implicated in the control of bodyweight. (**a**) *CellChat*-based cell-cell interactions. *Left*: The number of putative interactions between *Lxn*^+^ or *Lxn*^−^ astrocytes and neurons (*Pomc*^+^ or *Agrp*^+^) in control (normal chow) and after exposure to a high-fat hypercaloric diet (HFD)^37^. *Right*: Reconstruction of efferent (‘outbound’) and afferent (‘inbound’) signaling patterns across the four cell groups. (**b**) Changes in gene expression for mitochondrial genes upon exposure to HFD. (**c**) Correlated expression for *Lxn/Mfn2/Gfap* in astrocytes. (**d**) Illustration of astrocyte contributions to the control of synaptic communication. (**e**) GFP^+^ astrocytes after viral transduction (*AAV-Gfap-Gfp-Cre*) of the ARC in *Mfn2*^f/f^ mice. (**f**) Representative ultrastructural details of mitochondria in astrocytes under control conditions and the virus-mediated ablation of Mfn2. (**g-h**) Quantitative electron microscopy of morphological parameters in control vs. site-directed astrocyte-specific *Mfn2* loss-of-function. (**i,j**) Metabolic parameters in control vs. *Mfn2*^f/f^::*Gfap*^Cre^ mice under normal-fed *vs*. HFD conditions; experiments were performed in adult male mice at the age of 6–25 weeks. Data were expressed as means ± s.e.m.; sample size was at least 4 animals per condition; two-tailed t-test with Welch’s correction was used for statistical analysis: *P < 0.05, **P < 0.01 (**h-j**). *Scale bars* = 100 mm (**e**), 500 nm (**f**).

Lastly, we sought to address if astrocytes in the ARC could directly impinge upon neurons of the food intake circuitry to modulate body weight. Firstly, we examined if HFD affected the coupling of either *Lxn*^+^ or *Lxn*^−^ astrocytes to *Pomc*^+^ and/or *Agrp*^+^ neurons (Fig. 6a, **Extended Data Fig. 12,13**). HFD triggered cell type-specific rearrangements of signaling pathways in both astrocytes and neurons, including increased angiopoietin (*Angptl*), epidermal growth factor (*Egf*), and melanocortin signaling. Secondly, we determined if metabolic pathways were also altered in *Lxn*^+^ astrocytes upon exposure to HFD, and primarily found up- or down-regulated genes that affected mitochondria functions (Fig. 6b). Specifically, genes regulating mitochondrial fission (*Fis1, Dnm1, Dnm1l*, and *Mff*) were decreased. In contrast, mitofusins (*Mfn1* and *Mfn2*) were increased. Thirdly, we dissected a close positive correlation between *Lxn* and *Mfn2* expression, the latter controlling the fusion of mitochondria^94^. Moreover, HFD induced the expression of *Mfn* genes in astrocytes (see also: **Extended Data Fig. 1f**). Thus, we suggest a potential role for *Mfn*1/2 in astrocytes to affect energy homeostasis locally (Fig. 6c).

Considering that a HFD affected *Mfn1/2* expression, we posited that *Lxn*^+^/*Gfap*^+^ astrocytes could contribute to the regulation of food intake (by modulating POMC output)^95^, that requires periodic synaptic reorganization, a mechanism of high energy demand, and regulated by astrocytes (Fig. 6d)^82^. To test this hypothesis, we have generated astrocyte- and ARC-specific *Mfn2* knock-out mice by injecting AAV8-*Gfap-Gfp-Cre* particles in the ARC of *Mfn2*^f/f^ mice (Fig. 6e, **Extended Data Fig. 15**). Inactivation of *Mfn2* in astrocytes led to aberrant mitochondria, the loss of astrocyte ensheathing the perikarya of neurons, and a reduced number of synapses apposing POMC^+^ neurons (Fig. 6f–h). These changes were associated with a significant increase in food intake, weight gain, and increased fat accumulation in mice exposed to an HFD (Fig. 6i,j). Cumulatively, these data suggest that AstroTRAP can predict target genes in subsets of regionalized astrocytes that underpin specific metabolic functions by modulating the connectivity of neurons.

## Discussion

Astrocytes, as this study shows, are more heterogeneous than generally believed. Most notably, astrocytes under physiological conditions display fundamental topographical differences to allow their matchmaking with local neurons, for which astrocyte-driven metabolic support, signaling, and protection are minimally required for survival, synaptic neurotransmission, and resilience to external stressors. However, the molecular make-up of astrocytes is much more dynamic than that of neurons and can be portrayed as onion-skin-like layers, of which, only the outermost is taken into account by most contemporary research. Therefore, astrocytes could only be subdivided as healthy (‘resting’) or pathologically-transformed (‘reactive’, marked, e.g., by *Gfap* expression). The novelty of our study is to show that astrocytes exist in many stable cell-states, which we primarily subdivided as: *i*) a *‘metabolosyncratic’ ground state*, which refers to the expression of pleiotropic metabolic markers like, *Aqp4, Glul* to support general neuronal metabolism/survival and *ii*) *‘alloplastic’ astrocytes* that differentially express GRNs^3,13^ in anatomical foci to match and to alleviate allostatic pressure on neurons. These features include cellular checkpoints that prevent astrocytes from transforming into ‘reactive’ entities priming pathological states. Using its walk-back feature selection, AstroTRAP could differentiate gene sets and GRNs that otherwise overwrite and obscure key differences in genetic signatures specifying their spatial distribution, and conveying novel information on astrocyte-neuron interactions when organizing neuronal output. The view we advocate, therefore, is that astrocytes are integral to each neurocircuit, and work as signal detectors (through their receptor repertoire), excitability controllers (through ionostasis and tonic inhibition/excitation), metabolic organizers, and signaling nodes to instruct neurons (through a plethora of astrocyte-specific gliotransmitters and by supplying neurons with neurotransmitter precursors), thus being equal ‘partners’ to neurons in organizing environment-brain interactions, and generating neuronal output patterns for adequate behavioral outcomes.

Addressing astrocyte heterogeneity has so far eluded most, if not all, algorithms because these cells defy neuron-specific rules, by being exceptionally plastic at both the molecular and cellular (morphological and functional) levels. Yet, AstroTRAP was successful in ordering and stratifying single-cell RNA-seq data even from an unmarked mixture of cells in the hypothalamus, one of the functionally most diverse brain regions where neuroendocrine output is shaped by as few as 1,000–10,000 neurons under physiological conditions. Considering that the ratio of astrocytes and neurons varies widely across hypothalamic areas (from ~3:1 to ~1:3)^2^, we suggest that AstroTRAP can reach equal depth to ‘Hypomap’^6^ (or other neuron-oriented protocols) to resolve the functional diversity of the astrocyte lineage.

An unexpected finding of our study is that astrocytes show many of the zonal and regional gene markers that neurons harbor. This finding is, nonetheless, logical considering that most hypothalamic cells are generated in the proliferative zone of the wall of the 3^rd^ ventricle, where ependymocytes and tanycytes can equally give rise to neurons and astrocytes^78^. We find it exciting for future studies to test if the same domains of progenitor cells give rise to both neurons and astrocytes that co-populate particular areas of the hypothalamus, and whether neuronal and astrocyte progeny share migratory routes (although at different times), and co-evolve locally through a tightly-controlled bidirectional exchange of information by using defined (sets of) neuro- and gliotransmitters. Once mature, an individual astrocyte can connect to many neurons and neigbouring blood vessels. It seems logical, therefore, to next determine if perisynaptic leaflets and perivascular endfeet formed by astrocytes are as versatile, and functionally segregated as neuronal synapses. Although such studies will require a novel level of spatial resolution, their functional differences are supported by recent lineage barcoding studies^96^ that revealed a close molecular relationship between neurons and astrocytes co-populating and co-operating in specific brain areas.

An increasing number of experimental studies^70,97–99^ implicate astrocytes in the regulation of behavior. Our findings in the food intake circuitry are compatible with this concept. However, the thrust of or work is not only to link astrocytes to behavior but doing so through identifying site-specific candidate genes (*Mfn1/2*), thus linking these candidates to cellular plasticity. The subsequent use of loss-of-function experiments expanded the conceptual framework that the motility of perisynaptic leaflets form astrocytes both within the hypothalamus and at the median eminence^100^ are critical to determine synaptic coverage at the sites of hormone and/or neurotransmitter release. Any change in the astrocytic coverage of neuronal structures can only be at the expense of afferent synapses. Indeed, the loss of synaptic input onto POMC^+^ neurons, which drive satiety and limit over-eating^82^, can be interpreted to silence these neurons, thus shifting the output of the food intake circuitry towards promoting obesity at the organismal level.

Overall, our study provides both a tool to study astrocytes and a platform to develop novel hypotheses by means of cell-type-specific molecular predictors for neurophysiology and likely brain diseases.

## Online Methods

### The ‘AstroTRAP’ pipeline

#### Downloading and preprocessing single-cell RNA-seq data:

Raw sequencing data files were obtained from the NCBI Gene Expression Onimbus mirror of the European Bioinformatics Institute using the IBM Aspera tool-kit (example command: - *ascp -QT - l 800m -k 1 --overwrite=diff -P 33001 -i ~/asperaweb_id_dsa.openssh* era-fasp@fasp.sra.ebi.ac.uk*:/path/to/file/on/server*) or directly from the NCBI mirror using the ncbi-toolkit based on the BioProject ID (example command: esearch -db sra -query PRJNA****** | efetch -format runinfo | cut -d ‘,’ -f 1 | grep SRR | xargs -n 1 -P 20 prefetch --max-size u && esearch -db sra -query PRJNA****** | efetch -format runinfo | cut -d ‘,’ -f 1 | grep SRR | xargs -n 1 -P 20 fasterq-dump -p -x --threads 10 --mem 20000M -- outdir fastq --split-files --include-technical && pigz -p 20 fastq/SRR*.fastq). If an original bam-file from the 10X Genomics Cell Ranger count pipeline was deposited, it was further converted to fastq (example command: ~/src/cellranger-7.1.0/bin/cellranger bamtofastq --nthreads=12/data/PRJNA******/bam/SRR*******.bam/data/PRJNA******/fastq/SRR*******/). Information regarding publicly available data deposition are **in ED Table 1**.

#### Deriving initial UMI-count matrices:

The Cell Ranger pipeline (*v7.1.0*)^102^ was used for sample demultiplexing, barcode processing, and single-nucleus gene counting. Reads containing sequence information were aligned using the optimized mouse genome reference (*vmm10_optimized_v.1.0*) provided by Pool’s lab^46^, which is based on the default Cell Ranger mm10 genome version 2020-A that was cleared from gene overlaps, poorly annotated exons, 3’-UTRs, and intergenic fragments. PCR duplicates were removed by selecting unique combinations of cell barcodes, unique molecular identifiers (UMIs), and gene IDs. Thus, a gene expression matrix was created and used for further analysis. We aligned reads using ‘--include-introns’ quantification mode.

#### RNA-velocity for count matrices:

To obtain multiple count matrices for each read source (related to exon/intron gene structures), we have built a *piscem* index^103^ of the reference genome for the *spliceu version* of the *simpleaf* quantification pipeline (*vdocker://etretiakov/usefulaf:0.9.0*) based on alevin-fry^104^. This approach achieved the high-quality resolution of any ambiguity of the read source, which was particularly important in case of single-nuclei RNA-seq^103,105,106^, which can strongly affect the outcome of RNA-velocity estimates^107,108^.

### Preparation of individual single-cell RNA sequencing datasets for analysis

#### 1. Droplet selection:

The droplet selection method of Cell Ranger is based on the *EmptyDrops* method^109^ incorporated into the Cell Ranger count pipeline. We refer to **ED Table 1** for Information on the number of detected cells/nuclei.

#### 2. Ambient RNA removal:

We applied *CellBender*, a neural network-based approach (*docker://etretiakov/cellbender:v0.0.1*)^67^. To derive additional information on the degree of regionally different levels of contamination and the quality of particular samples, we set false positive rate thresholds at successive levels (0.1, 0.01, and 0.001) and powered the neural network to learn over 150 epochs with a total number of droplets included on knee plots. Our subsequent analysis was performed at all three resolutions and without correcting data variants. We refer to **ED Table 1** on the size of droplet sets used.

#### 3. Doublet detection:

For each sample, we separately quantified the probability of a cell being a doublet based on the expected doublet-rate reference table provided by 10X Genomics, and the number of cells/nuclei in the samples by Scrublet (*v0.2.3@pyh5e36f6f_1*)^68^. Information on the expected doublet rates used in this estimation are in **ED Table 1**. (*see also: GetDoubletRate* in *function.R* and *scrublet_cb.py* files of the code directory).

#### 4. Further filtering:

Information on gene annotation was added by using the *gprofiler2* package (v0.2.1)^69^. Accordingly, we filtered cells based on their high content of mitochondrial, ribosomal, or hemoglobin genes. Specific thresholds were chosen individually for each dataset (see: *GitHub Exploratory Analysis reports and params.json files*). Additionally, pseudogenes and poorly annotated genes were deleted from the count matrix. Cells of low complexity were filtered out, too (*as log_10 Genes/log_10 UMI*). Cells were then assigned cell cycle scores using the *CellCycleScoring* function in the Seurat package (v4.3.0)^110,111^. For correlation analysis of astrocytes in ARC^40^, we filtered cells based on the expression of 13 mitochondrially coded genes, using a strict approach to avoid bias caused by the technical variability of sequencing when exploring *Mfn2* in particular. To do so, we applied individual filtering models fitted for each sample of the dataset separately^112–114^ (for packages *splines, flexmix, miQC*): *536-1_chow-diet, 536-3_chow-diet, 537-5_538-2_high-fat-diet - mixtureModel with posterior cutoff 0.95; 536-5_chow-diet - spline mixtureModel with posterior cutoff 0.7, 537-1_537-3_high-fat-diet - spline mixtureModel with posterior cutoff 0.95, 536-2_537-4_high-fat-diet - spline mixtureModel with posterior cutoff 0.999999.*

### Astrocyte selection

The analysis described here were performed twice, with the 1^st^ iteration exploring all cell types, while the 2^nd^ focusing on particular glial cell types: *i*) astrocytes, *ii*) tanycytes, *iii*) ependymal cells and NG2-glia, and *iv*) oligodendrocyte precursors (ODPs).

#### 1. Gene selection:

The selection method of the Seurat package (v4.3.0)^110,111^, which uses a modern variance of stabilizing the transformation of statistical technics that utilizes the scaling to Pearson residuals^115^. Thus, we selected 3,000 highly variable genes *per* dataset, and regressed out complexity and cell-cycle variability prior to the final scaling of the filtered matrixes.

#### 2. Graph-based and multi-level reconcile tree clustering:

We performed Leiden algorithm graph-based clustering. PCA was performed using selected genes. Jackknife tested^116^ principal components (we tested the significance of feature for randomly picked in 100 samples by 2% of data, each over 1,000 iterations; see *PCScore function in the functions.R script of the code directory*) allowed us to construct a shared nearest neighbor graph by relying on the overlap amongst the 15 nearest neighbors of each cell. Leiden modularity optimization^117^ was used to partition this graph with an array of resolution parameters, where 30 modularity events were sampled between 0.2 and 2.5. Visualizations of clustering trees^118^ were produced using the clustree package (v0.5.0), which showed the resolution of previously identified clusters. By inspecting these resolutions, a reconciled tree was produced by the mrtree package (v0.0.0.9000)^119^, including a calculation of an adjusted multi-resolution Rand index, which was then chosen as the maximum value if there was no higher modularity within an additional 0.05 AMRI difference (see: *SelectResolution in the function.R file of the code directory*).

#### 3. Marker genes:

Marker genes for each cluster were identified using the *logreg* test^120^ implemented in the Seurat framework (v4.3.1)^121^. Genes were considered significant markers for a cluster if they had an FDR of < 0.001. Identities were assigned to each cluster by comparing the detected genes to previously published markers and our own validation experiments. We assigned the ‘astrocyte’ label to a cell only if it contained at least *n* = 7 marker genes. The list of *n* = 22 marker genes that we used as inclusion criteria are referred to in **ED Table 2** (see: *class_cello.py* files of the code repository).

#### Classification of cell types based on gene-scoring based on information from publicly available databases:

We further explored gene signatures using enrichment with over-representation analysis^122^. To this end, we used canonical markers from *PanglaoDB* with particular focus on *i*) astrocytes, *ii*) tanycytes, *iii*) ependymocytes and ODPs (see: *class_cello.py* file of the code repository).

#### Filtering criteria for astrocytes:

Astrocytes were filtered with cell quality markers listed in the code repository as individual *params.json* files for each dataset. Regardless of the dataset, and the selection method of astrocytes, we further filtered cells using a manually curated list of 59 marker genes for each major subtype of cells (glutamatergic, GABAergic and peptidergic neurons, OPCs, mature myelinating oligodendrocytes, pericytes, vascular muscle cells, macrophages, microglia, tanycytes, and ependymocytes. Thus, any astrocyte to pass our filter ought to contain a maximum of *n* = 2 genes from the list of exclusion criteria as strict trade-off, also taking into account the effect of ambient RNA (**ED Table 1**). Marker genes used as exclusion criteria were listed in **ED Table 2** (see: *class_cello.py* file of the code repository).

#### Prepare, train, and test data splitting to enable hyperparameter optimization for supervised machine learning models by classification performance metrics:

As we had variable numbers of input cells in subregional datasets, we defined the probability of a cell to be in the training set as 90%, or if this percentage was smaller than the ratio of 1,000 cells/the total cell number in the dataset. We used this probability to randomly assign cells to the training set. We used this process to obtain more balanced training sets regardless of the initial number of astrocytes in a hypothalamic region. We used the same training sets for all models to ensure that the results were comparable. The remaining cells were assigned to the test set to enable the evaluation of the model’s performance on unseen data. We used the same test sets for all models to ensure comparable results (see: class_cello.py file of the code repository). As a result of the previous steps, we derived subsets of astrocytes from each of 12 individual subregional dataset, split into train/test sets and 4 ‘whole hypothalamus’ datasets (**ED Fig. 4a/Step 0**).

### Identification of astrocyte signatures and diversity description across hypothalamic subregions

#### Full conventional integration:

We first performed data integration using well-established anchor-based integration using the Seurat package (v4.3.0) to compare astrocytes from different hypothalamic areas^110,121^. We used both *SCTransform*-based and *GLM-*scaled versions of the default pipeline aimed to explore astrocyte heterogeneity with varying numbers of highly variable genes selected (2,500—7,500; initial *hvg param*). A particular problem we have encountered was the shallow fraction of variability shared across all analyzed hypothalamic regions (no more than 426 genes), which mostly were general astrocyte markers common across the nervous system.

**Differential gene expression** **(DGE)** analysis on *SCTransform*-corrected UMI-count matrices was statistically evaluated to obtain differentially expressed genes using log-normalized values with pseudocount = 1 for *n* = 12 regional hypothalamus clusters using the MAST test^123^, as described^115,124^. Results were provided in **ED Table 3**.

#### Iterative paired integration while masking shared cellular signatures:

We used an alternative strategy in which shared signatures of astrocytes were reduced to address if any level of regional heterogeneity could be uncovered behind astrocytes transiting across functional states. To avoid that rapidly reducing the number of anchor genes used to integrate datasets would become a limitation of precision, we have preferred the pair-wise integration of each subregional dataset with each ‘whole hypothalamus’ dataset using Python implementation of the Harmony algorithm^73^. To define regional differences amongst astrocytes, rather than functional state-changes (*as above*), anchor genes (defined as a subset of highly variable features corrected for concatenated datasets to maximize the stringency of a variance-stabilizing transform procedure^125^ optimized on the earlier pair-wise integrations) were extracted, with 48 sets of 2,000 genes in total or 12 sets (each for a hypothalamic nucleus)^37,40–42,46–52^ per a reference set of the ‘whole’ hypothalamus^7,43–45^ (see: *get_full_pair_mtx.py* file of the code repository; PaCMAP embedding of integrated datasets before ambient RNA removal as in **ED Fig. 4a/Step 1**). Thus, a list of genes ordered by their descending variance on the same plane of the whole hypothalamus reference was extracted and adapted to a rank aggregation algorithm^126^ to distinguish broadly-expressed genes associated with functional cell-states: *i*) we derived scores per a ‘whole’ hypothalamus reference dataset, accounting for genes in the mouse genome version as above (45,163 genes in initial matrixes; see: *get_shared_signature.R* file of the code repository; **ED Fig. 4a/Step 2**). *ii*) We estimated rank aggregation scores of 4 derivate lists (ED Table 3; see: get_aggregated_shared_signature.R file of the code repository; **ED Fig. 4a/Step 3**).

Consequently, we derived integrated manifolds between 12 area-specific^37,40–42,46–52^ and four ‘whole’ hypothalamus datasets^7,43–45^, again after removal of shared signatures using *n* = 100, 250, and 500 genes as thresholds (redundant correlated genes were also removed by absolute Pearson’s correlation with> 0.5 corresponding to each threshold; **ED Fig. 4a/Steps 4–5**) to estimate the impact of function-specifying genes (see: *get_substr_pair_mtx.py* file and particularly *get_top_abs_correlations()* and *remove_irrelevant_top_abs_correlations()* functions in the code repository; **ED Fig. 4a/Steps 6–7**). These steps were used to *i*) unmask potentially hidden spatial marks in astrocytes from different hypothalamic nuclei, *ii*) clean the genes axis of the expression matrices, and *iii*) have their respective manifolds dimensionally reduced by the integration.

The downstream analysis of the subregional heterogeneity of astrocytes reported here used datasets after removal of ambient RNA at 0.001 FDR, as well as of a shared signature of *n* = 100 genes (as threshold) with correlated genes as described above, and using *k* = 10 to construct graphs for nearest neighbors (NN; ks = k + 10 = 20 for expanded mutual kNN-graphs with a path-connectivity model) as our limited testing (*FDR=[0.001, 0.01, “nc”]; signature_substruction=[100, 250, 500, “full”], neighbors_k=[5, 10, 25, 50], connectivity_mst=[“full_tree”, “min_tree”]*) demonstrated best performance. We also had to reduce complexity (see S*nakefile* in the root of the main GitHub repository). To explore relevant parameters combinations, we used a simplified approach: *i*) we performed over-clustering of the training set of each integrated pair using clustering from *leidenalg* (v0.9.1@pypi_0)^117^, *ii*) subset each of the 12 matrices to the same reference dataset^43^, *iii*) derived a fuzzy simplicial set using UMAP-based path-connectivity^127,128^, *iv*) aggregated results (microclusters and graphs) by partitioning-based graph abstraction^129^, *v*) PAGA graphs were utilized as layers of multiplexed graphs to optimize multiplex partitions^117,130^, *vi*) used those partitions to train a support vector machine (SVM) classifier (SVC)^131,132^ and to project those partitions on test sets astrocytes, and vii) embedded a full reference dataset labels for supervised densMAP^133^ data embedding using derived (see: *microclustering_limited_test.ipynb* file of the analysis repository; an example of unfiltered (*FDR=“nc”, signature_substruction=“full”, k=10, ks=20, connectivity_mst=“min_tree*”) (Fig. 1K,M).

### Area-specific hypothalamic astrocytes revealed by AstroTRAP

#### Feature selection in 2 steps:

We prefiltered features using methods to capture genes of known subregional origin (**ED Fig. 4a/Step 11**). To this end, we used a union of gene sets selected by c^2^ statistics^75^, ANOVA *F*-value^76^, and mutual information score^77^. We coincidently used these feature selection methods to improve the accuracy and stability of the selected features, leading to better performance in machine learning models. We used *GridSearchCV* to optimize parameters of the pipelines that consisted of 3 steps: ‘scaling’: *MinMaxScaler* for consistency (as negative values were not allowed in our calculations), ‘reduce dimensionality’: *SelectKBest* taking input one of c^2^ (*f_classif or mutual_info_classif*), and ‘classify’: SVC estimator with scaled gamma and a maximum number of iterations fixed to 1,000. The parameter grid included SVM fit strength (*C_OPTIONS = [1, 10, 100, 1000]*), and a number of features (‘K’) to select with genes thresholds (2,000, 1,000, 500, 250)^134^. Then, we used *RepeatedStratifiedKFold(n_splits=10, n_repeats=10)* cross-validation to select classifiers with the highest weighted F-measure scores. Finally, we extracted features from each *best_estimator* and merged them into one set of genes (**ED Fig. 4a/Step 11; ED Table 4**). The union of feature sets allowed us to filter input to the downstream pipeline.

#### Best SVM classifier model with *ad hoc* feature selection for region-label assignment using Grid-Search grouped k-fold cross-validation:

To build an effective classifier model that reconciles features of subregional origin regardless of functional cell-states (that were captured as shared signatures), we used astrocytes pre-selected from area-specific datasets (**ED Fig. 4a/Step 0**), and pre-filtered gene sets (**ED Fig. 4a/Step 11**). To learn features that consistently represent regional heterogeneity, we recognized stand-alone functional roles of nuclei/areas of the hypothalamus. Functional state-differences amongst astrocytes might occur across the whole hypothalamus due to environmental and/or neuronal influences. Thus, we hypothesized that astrocytes are not specific to a given region at the long term; instead undergo continuous complex trajectory transitions. To resolve these problematics, we clustered their shared structure into tiny groups of cells that were being examined separately. Thus, we inferred a reduction in intra-regional specificities and could focus on inter-regional characterization at the transcriptome (gene) level. After having reduced number of genes and meaningful groups for stratification, we applied feature selection pipelines from ‘scaling’ *RobustScaler* that smooth outliers, feature selection, and classification by SVC. We implemented two types of feature selection:*LogisticRegression* that utilized L1-regularisation to fit coefficients for a multiclass problem vs. rest (for the sake of computational performance in contrast to multinomial estimation); and the *XGBoost* classifier. To fit these models, we encoded region classes as sorted proportions of each class. To select the best model, we performed *GridSearchCV* with *StratifiedGroupKFold(n_splits=10)* cross-validation on the training set scored by the Matthews correlation coefficient (MCC)^135^, as regional class proportions were unbiased.

#### Identifications of astrocyte clusters beyond functional states:

To further consolidate the signal we have gained, graph representations were generated using a minimal spanning tree also implementing A path-connectivity model of the UMAP algorithm^136^.Firstly, we reduced 12 integration manifolds of training data^43^, using the UMAP algorithm^128^ with following parameters: *n_components*=6, *n_neighbors*=20, *n_epochs*=1000, *metric*=“cosine”, *init*=“spectral”, *learning_rate*=0.1, min_dist=1, *spread*=2, *repulsion_strength*=2.0, *negative_sample_rate*=10, *angular_rp_forest*=True, and *densmap*=False. Secondly, the UMAP algorithm drove the 10-NN graph from this matrix in Euclidean space with pynndescent optimization. Thirdly, a 20-NN graph and distance matrix was used to build a 20-mutual-NN graph using the minimal tree path-connectivity model (functions: *min_spanning_tree(), create_connected_graph(), find_new_nn(), mutual_nn_nearest() in microclusters_to_groups_cv_feature_selection_svc.ipynb*)^127^. Fourthly, we derived a fuzzy simplical set for the ensuing graph using the UMAP algorithm^136^. Graph information were saved in an *anndata* container by being transformed *from_scipy_sparse_array* to *iGraph*, and through *igraph.Graph.get_adjacency_sparse()* to *adata.obsp[“connectivities”]* object. We also derived the required *adata.obsp[“distances”]* using the *get_sparse_matrix_from_indices_distances_umap()* function (see: *microclusters_to_groups_cv_feature_selection_svc.ipynb* file in the main GitHub analysis repository; **ED Fig. 4a/Step 8**)^137–139^. Next, we used *Optimiser* class from the *leidenalg* package to explore the resolution profile of the Constant Pots Model clustering on the weighted graph at a range from 0 to 2, until full convergence^117^. Thus, we partitioned the weighted graph using the *CPMVertexPartition* function with *resolution_parameter=0.0112*, and applied the *optimise_partition* function of the optimizer until convergence^117^. The partitioning membership was saved as a categorical vector to the *PRJNA779749_init.obs[“subfunct_groups”]* (**ED Fig. 4a/Step 9**). Thus, we trained 12 support vector machine classifiers (*linear kernel, svm fit strength - C=100*) from the *sklearn* package to fit corresponding labels (*PRJNA779749_init.obs[“subfunct_groups”]*) to the subsets of 12 integrated matrices. Therefore, we projected learned group labels to the corresponding regional parts of the training sets (**ED Fig. 4a/Step 10**).

#### Optimization of hyperparameters for a logit-SVM classifier model:

We trained our pipeline with *SelectFromModel(LogisticRegression(solver=“saga”, multi_class=“ovr”, penalty=“l1”, max_iter=10000, n_jobs=−1,), threshold=−np.inf)* feature selection and SVC estimator with scaled gamma and a maximum number of iterations fixed at 10,000. The parameter grid included SVM fit strength (*C_OPTIONS = [1, 10, 100, 200]*) and maximal number of features to select with *N_FEATURES_OPTIONS = [round(n_features / 3), round(n_features / 4), round(n_features / 8), round(n_features / 10), round(n_features / 20)]* (where *n_features* equals the number of genes in the union of prefiltered sets). We have also extracted features from *best_estimator* (**ED Table 4**). The best model was then trained on the whole training set, and then stored (**ED Fig. 4a/Step 12**).

#### Optimization of hyperparameters for the XGBoost-SVM classifier model:

Our pipeline was trained with SelectFromModel(XGBClassifier(tree_method=“gpu_hist”), threshold=−np.inf) feature selection^140^ and SVC estimator with scaled gamma and a maximum number of iterations fixed at 10,000. The parameter grid included SVM fit strength (C_OPTIONS = [1, 10, 100, 200]). At the feature selection step, we have optimized parameters of the estimator as learning_rate”: [0.1, 0.3, 0.5], boosting trees depth and number of rounds at max_depth: [3, 5, 7], n_estimators”: [100, 250, 500], and the maximal number of features to select as N_FEATURES_OPTIONS (see logit-SVC section). We have also extracted features from *best_estimator* (**ED Table 4**). Finally, we trained the best model on the whole training set, and stored it for further evaluation (**ED Fig. 4a/Step 12).**

#### Evaluation of the best classifier model:

Based on the above optimization, we have built two SVM-based classification pipelines, used on our test dataset with additional validation on ‘whole’ hypothalamus predictions: *i*) *LogisticRegression (logit*)-based feature selection with L1-regularisation + SVC, which identified 1,406 genes during the feature selection stage (**ED Table 4**), and performed with 0.441 MCC and 0.627 weighted *F*-measure on the evaluation set. During cross-validation, the mean test MCC score = 0.619 (**ED Table 5**). *ii*) *XGBoost*-based feature selection + SVC, which returned 211 genes, when *learning_rate*=0.3, boosting trees *max_depth*=3, and number of rounds *n_estimators*=100 for feature selection, and a strong (C = 200) SVM classifier (**ED Table 4**). This algorithm outperformed the simpler *logit*-SVC model with 0.578 MCC and 0.723 weighted F-measure on the evaluation set (Fig. 2a; **ED Fig. 5a**; during cross-validation, the mean test MCC score = 0.871; **ED Table 5**). These models were used to project learned regional differences onto ‘whole’ hypothalamus datasets^7,43–45^. To do so, we used the original model to select directly from the whole set of available genes from the regional dataset matrices. Thus, our models were re-fitted to a subset of genes available for prediction in a particular dataset, resulting in 4 different models/pipelines. Given these constraints, we have initiated an *ad hoc* approach to save processing time and to limit interpretation, resulting in a shared model across each predicted dataset. Herein, *we i*) selected subset genes that were expressed in more than 10 cells in each ‘whole’ hypothalamus dataset, *ii*) took the intersection of these genes across the 4 reference datasets. This resulted in 2 best-models selected by cross-validation: a) fit strength C = 10 and 1,378 genes (**ED Table 4**) that were used for the *logit*-SVC model, which performed with a mean test MCC score of 0.714; b)fit strength C = 200, *learning_rate*=0.5, boosting trees *max_depth*=3 and number of rounds *n_estimators*=100, returning 207 genes for the *XGBoost*-SVC model (**ED Table 4**), which performed with a mean test MCC score of 0.770 (**ED Table 5**). We used these final models to predict subclasses of astrocytes that could be spatially segregated and be present in each ‘whole’ hypothalamus dataset. On our evaluation set, *ad hoc* version models performed: *i*) 0.539 MCC and 0.703 weighted F-measure for the logit-SVC model, and 2) 0.518 MCC and 0.686 weighted *F*-measure for the *XGBoost*-SVC model. Spatially restricted modalities of astrocytes predicted by these direct versions of AstroTRAP (**ED Fig. 4A/Step 13**) were histochemically validated and functionally interrogated experimentally in cultured cells and *in vivo*.

### Functional annotation and validation of the regional heterogeneity of astrocytes

#### Gene regulatory networks (GRN) analysis by SCENIC:

We used SCENIC to confirm the GRN-driven heterogeneity amongst astrocytes, at a resolution that distinguished subgroups within distinct hypothalamic areas (Fig. 2B,C; **ED Table 6**). In doing so, we used normalized expression matrices of astrocyte subsets from ‘whole’ hypothalamus reference datasets, and then applied the SCENIC pipeline^79^ (docker://*aertslab/pyscenic:0.12.1*). to infer GRNs and their activity scores. For the *aucell* procedure, we have built GRNs only using enriched motifs with a NES of ^3^ 3.0. We then took only directly annotated transcription factors (TFs) or TF annotated for an orthologous gene into account and retained GRNs with ^3^ 3 genes. We used the *aucell* matrix to visualize dotplots of GRN activity, and to build a dendrogram by using Ward clustering^141^ (Fig. 2B; **ED Fig. 5**).

#### Ligand-receptor expression pairs between astrocytes and neurons:

To validate the regional specificity of *Lxn*^+^ / Insr^+^ astrocytes, astrocyte-driven intercellular signalling cascades were selected, and estimated using *CellChat*^86^, *Liana-py*^87^, and a rank aggregation method (including *CellPhoneDB*^142^, *CellChat*^86^, *ICELLNET*^143^, *connectomeDB2020*^144^, and *CellTalkDB*^145^). To provide a more accurate and transparent estimate of effect sizes and their confidence intervals, the Data Analysis with Bootstrap-coupled ESTimation (DABEST)^88^ was conducted examining the cumulative effect of cell-to-cell communications between Lxn^+^ and Lxn^−^ or Insr^+^ and *Insr*^−^ astrocytes and neurons in different physiological states. Therefore, the data are cleaned by filtering based on p-values, retaining only results with a significance threshold of less than 0.05, then merged into a single dataset, with additional categorical variables added to denote the region, condition, and control status. We included all ligand-receptor pairs regardless of their presence in each dataset or unique. Splitting the data into multiple groups and performing unpaired comparisons of the magnitude and specificity rankings between groups we produced Gardner-Altman plots of mean difference and Cohen’s d effect size, which provided a clear visualization of the differences between groups^88^. The analysis results conducted using CellChatDB^86^ only or all databases pulled by Liana-py^87^ showed consistent effects, so we preferred using CellChatDB as a manually curated one.

#### Sample sizes, statistics, and reproducibility for ‘AstroTRAP’:

Comparisons of model performance for hyperparameter optimization during grouped 10-fold cross-validation were addressed using *GridSearchCV*. The performance of models was evaluated using *matthews_corrcoef* (MCC). The output of *GridSearchCV* did not provide information on the certainty of the differences between the models. Therefore, we have conducted statistical tests on MCC results. Several variance-corrected statistical tests have been developed to meet these demands. We used the Nadeau and Bengio’s corrected *t*-test^146^ to obtain the highest replicability scores (which rate how similar the performance of a model is when evaluating it on different random partitions of the same dataset), while maintaining the lowest possible rate of false positives and false negatives. We implemented this analysis under both frequentist and Bayesian statistical frameworks. Herein, testing assumed a corrected right-tailed paired *t*-test to evaluate if the performance of the 1^st^ model was superior to the 2^nd^ model, where our null hypothesis was that the 2^nd^ model performed at least as good as the 1^st^ model. Moreover, we used Bayesian estimation to calculate the probability that the 1^st^ model is better than the 2^nd^. Bayesian estimation provides a distribution followed by the mean μ of the difference in the performance of two models. To obtain the posterior distribution, we assumed a *priori* how the mean might be distributed before working with the actual data and multiplied those by a likelihood function that computes how likely our observed differences were given the values that the mean of differences could take. One way to define posterior distribution is by using a closed-form expression to select a prior conjugate to the likelihood function. Benavoli *et al*.^147^ showed that, when comparing the performance of two classifiers, one can model the prior as a normal-gamma distribution (with both mean and variance unknown) conjugated to a normal likelihood, thus expressing the posterior as a normal distribution. Marginalizing the variance from this normal posterior, the posterior of the mean parameter can be defined as Student’s *t*-distribution. Overall, we computed the probability that a model performs better, worse, or quasi-equivalent to another using the Bayesian approach.

DGE of the assumed regionalized subclasses of astrocytes projected onto ‘whole’ hypothalamus datasets and UMIs counts matrices were statistically tested to obtain DEGs for *n* = 8 available hypothalamic territories using the *MAST* test^123^. We used the *Logreg* test^120^ to define DGE across cells tentatively assigned to projected hypothalamic areas on ‘whole’ hypothalamus reference datasets. Finally, we applied rank aggregation of the DEGs across the different datasets to derive individual lists of markers for each nucleus^126^. We targeted RRA on the *p*-value to determine the specificity score and on the log_2_ fold change to determine the magnitude score (**ED Fig. 4A/Step 14**; Fig. 2A–F; **ED Table 6**).

#### Other packages:

Visualizations and figures were created in the *ggplot2* (v3.4.2), *cowplot* (v1.1.1)^148^, *patchwork* (v1.1.2.9000), and *scCustomize* (v1.1.1) packages using the viridis color palettes (v0.6.2) for continuous data. *UpSet* plots^149^ were produced using the *UpSetR* package (v1.4.0)^150^ with help from the *gridExtra* package (v2.3)^151^. Data manipulation was performed using *tidyverse* (v2.0.0.9000), particularly *dplyr* (v1.1.2), *tidyr* (v1.1.2), and *ork* (v1.0.1)^152^. Analysis was managed in the *Snakemake system* (v7.21.0)^153^, and *orkflow* (v1.7.0)^154^, which was also used to produce the publicly available website for the analysis code, results, and output. Reproducible reports were produced using Quarto (v1.2), knitr (1.42)^155^ and *R Markdown* (v2.21)^156^, and converted to HTML using *Pandoc* (v2.19.2).

### *In vivo* and *in vitro* studies:

#### Ethical approval of animal studies:

Experiments on live animals conformed to the 2010/63/EU European Communities Council Directive and regulated by applicable local laws (Tierversuchsgesetz 2012, BGBI, Nr. 114/2012, Austria). Experimental protocols were approved by the Austrian Ministry of Science and Research (66.009/0145-WF/II/3b/2014, and 66.009/0277-WF/V/3b/2017). Feeding experiments using special diets were approved by the Institutional Animal Care and Use Committee of Yale University and performed in its Animal Resources Center. Particular effort was directed towards minimizing the number of animals used, and their suffering during experiments.

#### Mouse strains:

Mice were group housed (*n* = 3 – 5) at 22 C° – 24 C° using a 12-h light/12-h dark cycle. Animals had *ad libitum* access to water and specified diet at all times. Postnatal animals were weaned on postnatal day (P)21. Mouse strains were commercially available, as follows: C57Bl/6J (‘wild-type’, RRID:IMSR_JAX:000664), Ai6 (RRID:IMSR_JAX:007906), Ai14 (RRID:IMSR_JAX:007914), *Pomc–gfp* (RRID:IMSR_JAX:009593), *Mfn2*^f/f^ (RRID:IMSR_JAX:026525), TRAP1 (RRID:IMSR_JAX:021882), TRAP2 (RRID:IMSR_JAX:030323). Experiments were not randomized, neither were the investigators blinded to the experimental variables and assignments.

#### Stress experiments in TRAP mice:

Experiments were performed as previously described^157^. Briefly, tamoxifen injection (150 mg/kg, *i.p*.) in TRAP1 mice^157^ was followed by the injection of 4% PFA (in 50 μl physiological saline) in the right hindpaw 24h later. After a lag-time of 72 h to allow for ZsGreen expression in ‘stress-responder’ cells, mice were transcardially perfused as below.

#### Circadian activity of astrocytes *in vivo*:

TRAP2 mice^158^ crossed with Ai14 reporter mice were injected with 4-OH-tamoxifen, which allowed for tissue collection in a narrow time window to assess *Fos* expression. 4-OH-tamoxifen was injected during the light (CT6) and dark (CT18) periods, with tissues collected 6h later.

#### Tissue collection and fixation:

Animals were transcardially perfused with 50–100 ml of a fixative composed of 4% paraformaldehyde (PFA) in 0.1M phosphate buffer (PB; pH 7.4), the brains dissected out, and post-fixed in the same fixative overnight. Alternatively, animals were deeply anesthetized (5% isoflurane (AbbVie) in 1L/min air flow), their brains rapidly dissected, and immersion-fixed in 4% PFA in phosphate-buffered saline (PBS, 0.05M, pH 7.4) at 4 °C for 24 h. Tissues were then extensively washed in PB, cryoprotected by immersion in 30% sucrose in distilled water at 4 °C 24–48h, and processed as described below.

#### Tissue preparation and histochemistry:

Free-floating sections were cut in 1-in-6 series at 50 μm thickness on a cryostat microtome. Glass-mounted serial sections had a thickness of 16 μm. After rinsing in 0.1M PB, specimens were exposed to a blocking solution composed of 0.1M PB, 10% normal donkey serum (NDS), 5% BSA, and 0.3% TX-100 for 3h. This was followed by incubation (for 48h) with select combinations of primary antibodies: goat anti-GFP (1:1,000; Abcam, #ab6662, RRID:AB_305635: lot GR311622-15, GR311622-7), chicken anti-GFP (1:500, Aves Labs, #GFP-1020, RRID:AB_10000240: lot GFP697986), chicken anti-mCherry (1:1,000; EnCor Biotechnology, #CPCA-mCherry, RRID:AB_2572308: lot 7670-4), guinea pig anti-GFAP (1:500; Synaptic Systems, #173 004, RRID:AB_10641162: lot 2–15, 2–17), rabbit anti-LXN (1:400; Sigma-Aldrich, #HPA014179, RRID:AB_1853404), rabbit anti-S100b (1:4,000; Synaptic Systems, #287 003, RRID:AB_2620024), mouse anti-NEUN (1:1,000; Millipore, #MAB377, RRID:AB_2298772), rabbit anti-AQP4 (1:4,000; Sigma-Aldrich, #A5971, RRID:AB_258270). After rinsing, tissue sections were exposed to cocktails of secondary antibodies (all from Jackson ImmunoResearch), including Alexa Fluor 488-AffiniPure donkey anti-goat (705-545-147, lot 131669), Alexa Fluor 488 donkey anti-mouse (715-545-151, lot 127820), Alexa Fluor 488-AffiniPure donkey anti-guinea pig (706-545-148, lot 138058), Alexa Fluor 647-AffiniPure donkey anti-guinea pig (706-605-148, lot 135631), Alexa Fluor 647-AffiniPure donkey anti-rabbit (711-605-152, lot 127614), carbocyanine (Cy)2-AffiniPure donkey anti-rabbit (711-225-152, lot 139999), Cy3-AffiniPure donkey anti-chicken (703-165-155, lot 142225), Cy3-AffiniPure donkey anti-goat (705-165-147, lot 134527), Cy3-AffiniPure donkey anti-guinea pig (706-165-148, lot 134844), Cy3-AffiniPure donkey anti-mouse (715-165-150, lot 116881), and Cy3-AffiniPure donkey anti-rabbit (711-165-152, lot 141941), that were applied at a dilution of 1:300 in 0.1 MPB supplemented with 2% BSA (20–22 °C, 2h). Nuclei were routinely counterstained with Hoechst 33,342 (1:10,000; Sigma). Tissues were coverslipped with entellan in toluene (Merck). Images were acquired in the ZEN2010 software package on a Zeiss LSM880 laser-scanning microscope equipped with appropriate excitation and emission filters for optimal signal separation.

#### Fluorescent *in situ* hybridization (HCR 3.0):

Multicolor stainings were performed on fresh-frozen tissues sectioned at 16 μm following the HCR v3.0 protocol for ‘*generic sample on the slide*’ (Molecular Instruments). Pre-treatment of tissue sections included, sequentially, fixation with 4% PFA for 15 min, 2x washing steps with PBS, and dehydration in an ascending EtOH gradient (25%, 50%, 75% and 100%, each step for 5 min with subsequent drying for 15 min). Tissues were obtained from 8–12 weeks old wild-type mice. Hybridization probes (*Apoe*: NM_009696.4; *Gfap*: NM_001131020; *Slit2*: NM_001291227.2; *Aldh1a1*: NM_001361503.1; *Tafa1*: NM_182808.3; *Plcb1*: NM_001145830.1; *Sgcd*: NM_011891.5; *Slc38a1*: NM_001166456.1; *Fos*: NM_010234.3; *Gja1*: NM_010288.3; *Snap25*: NM_011428.3; *Olig1*: NM_016968.4; *Htra1*: NM_019564.3; *Lxn*: NM_016753; *Adcyap1r1*: NM_007407.4; *Npy1r*: NM_010934.4; *Trhr*: NM_013696.2; *Tacr1*: NM_009313.5; *Grpr*: NM_008177.3; *Hcrtr2*: NM_001364551.1; *Prokr2*: NM_144944.3; *Otp*: NM_011021.5; *Isl1*: NM_021459.4; *Tbx3*: CCDS 19614.1; *Nr4a1*: CCDS 27848.1; *Foxg1*: NM_005249.5; *Pitx2*: NM_001042504.2) were purchased from Molecular Instruments.

#### Preparation, purification, and pharmacological probing of primary astrocytes:

Astrocytes were isolated from P6-P7 wild-type mice, which were anesthetized with 5% isoflurane (AbbVie, 1L/min flow rate), decapitated, their brains rapidly extracted and pooled in ice-cold Hanks′ Balanced Salt Solution (without Ca^2+^, Mg^2+^; Thermo Fisher). The meninges were peeled away with a fine forceps (Dumont No. 5; FST), and the hypothalamus was isolated by a curved-blade surgical scalpel. Previous protocols^159^ were then adapted to prepare high-purity cultures. Tissues were transferred into astrocyte growth medium, composed of Dulbecco’s Modified Eagle Medium (DMEM, high glucose, GlutaMAX^™^ supplement; Thermo Fisher), 10% fetal bovine serum (FBS, Gibco), and 1% penicillin/streptomycin (100 U/ml; Thermo Fisher), triturated, and centrifuged at 1,100 rpm for 5 min. After discarding the supernatant, tissues were digested in DMEM (with high glucose and GlutaMAX^™^; Thermo Fisher), 0.4% trypsin (Thermo Fisher) and 5% DNAse (Thermo Fisher) at 37 °C for 5 min. After terminating the reaction with 2% BSA, tissues were triturated with a serological pipette 3–4 times. Single cell suspension was generated by repeated washes, trituration, and 3 rounds of centrifugation (1,100 rpm, 3 min, at 22–24 °C). The resulting suspension was passed through a cell strainer (40 μm pore size, Fischer Scientific), washed, centrifuged, resuspended, and plated in astrocyte growth medium in T-75 flasks (10^6^ cells; VWR). Cells were cultured in astrocyte growth medium at in 5% CO_2_ atmosphere at 37 °C. Medium was partially (1/3) replaced every other day. At ^3^90% confluency, flasks were placed on an orbital shaker at 210 rpm at 37 °C overnight, thus separating neurons, microglia, and oligodendrocytes from adherent astrocytes. After discarding the supernatant, astrocytes were rinsed with Hanks’ Balanced Salt Solution (HBSS (+); with Ca^2+^, Mg^2+^; Thermo Fisher), and detached from the flasks’ surface with dissociation medium (0.25% trypsin; Thermo Fisher). Purified astrocytes were subcultured for 2–7 days, and then seeded (either for immunocytochemistry at 5 × 10^4^ cells/well in poly-D-lysine (Merck)-coated 24-well plates (VWR) containing 12-mm round coverslips (Carl Roth) or 3 × 10^5^ cells/well in 6-well plates (VWR) for biochemistry), and used for experiments. Cell pellets and supernatants were simultaneously processed, when needed.

The purity of astrocytes was controlled by immunocytochemistry. In brief, cells were washed 3x with ice-cold PBS (pH 7.4, Life Technologies) and fixed with 4% PFA (in 0.05M PBS; Sigma) at 22–24 °C for 10–15 min, and permeabilized by using 0.2% Triton X-100 (Sigma), 10% normal donkey serum (NDS; Jackson ImmunoResearch), 5% BSA (Sigma) in 0.05M PBS) at 22–24 °C for 60 min. After rinsing, cells were stained by a mixture of antibodies, including rabbit anti-ALDH1L1 (1:4,000; Synaptic Systems, #278 102, RRID:AB_2884922; for astrocytes), guinea pig anti-GFAP (1:500; Synaptic Systems, #173 004, RRID:AB_10641162: lot 2–15, 2–17; for astrocytes), goat anti-IBA1 (1:500; Abcam, #ab289874, RRID:AB_2942069; for microglia), rabbit anti-OLIG2 (1:200; Millipore, #AB9610, RRID:AB_570666; for oligodendrocytes), mouse anti-NeuN (1:1,000; Millipore, #MAB377, RRID:AB_2298772; for neurons) diluted in 0.2% Triton X-100, 5% NDS, 2% BSA in 1X PBS at 4°C overnight. Controls were carried out under identical conditions except without primary antibodies. After repeated rinses in 0.05M PBS, the specimens were exposed to species-specific secondary antibodies (Alexa Fluor 488- or carbocyanine (Cy)2/3/5-tagged (all 1:300; Jackson ImmunoResearch) diluted in 0.05M PBS, 2% BSA and Hoechst 33,342 (1:10,000 (Sigma); nuclear counterstain) at 22–24 °C for 2h. After another was in 0.05M PBS, coverslips were dipped in distilled water, mounted with glycerol (30 μL/coverslip), and then inspected by using a Zeiss LSM880 laser-scanning microscope. Emission spectra for each dye were limited as follows: Cy2 (505–530 nm), Cy3 (560–610 nm), and Cy5 (650–720 nm). Images were acquired in the ZEN2010 software package. Each coverslip was tested at 5 independent (random) fields of view, with the means ± s.d. of the cell counts from at least 4 cultures processed to establish a ‘purity index’ (that is the percentage of GFAP^+^ or ALDH1L1^+^ cells/total cells). Cultures were only processed with purity ^3^95%.

#### Cell viability assay:

The viability of cells, plated at a density of 25,000 cells/well in 24-well plates (VWR), was tested using the 3-(4,5-dimethylthiazol-2-yl)-2,5-diphenyltetrazolium bromide (MTT) assay^160^. In brief, growth media was replaced with 0.25 mL of MTT tetrazolium solution (1 mg/mL MTT tetrazolium (Abcam)), and exposed for 30 min at 37 °C. Subsequently, the incubation medium was replaced by 0.25 mL dimethyl-sulfoxide (Sigma), and the absorbance of the lysates was measured spectrophotometrically at 570 nm. Data in this report are from n = 8 independent observations (**ED Fig. 14**).

#### Latexin release from astrocytes:

Confluent cultures of astrocytes (in 6-plate format) were starved in serum-free growth medium overnight. Subsequently, the medium was replaced with either HBSS (−), ATP (100 μM; Sigma), or HBSS (+) for 5 min. To determine if insulin alters latexin release, cultured astrocytes were exposed to 0.05 μM, 0.1 μM, 0.3 μM, or 1 μM insulin (Sigma) that had been dissolved in serum-free growth medium for 30 min. Experiments were performed in triplicate. Subsequently, cell lysates, as well as protein fractions of the media were probed for latexin by Western blotting.

#### Quantitative Western blotting with total protein normalization:

Samples (cell lysate, medium and cell fractions) were obtained as described above. Western blot analysis was carried out using an Amersham WB System (GE Healthcare, Little Chalfont, UK).

Samples were labelled with carbocyanine (Cy)5 dye reagent (GE Healthcare; prediluted in ultrapure water (1:10); for total protein staining), reduced and boiled (at 95°C for 3 min) in sample buffer prior loading onto Amersham WB 13.5% gel cards (GE Healthcare, Little Chalfont, UK). Electrophoresis (600 V, 42 min) and protein transfer onto polyvinylidene-difluoride (PDVF) membranes (100 V, 30 min) were performed according to manufacturer’s instructions. After 1h of blocking in 3% BSA (Sigma) in standard Tris-buffered saline (TBS), membranes were incubated overnight at 4°C in primary antibody solutions against LXN (1:200: goat, ThermoFisher Scientific, #PA5-18676, RRID:AB_10980158). Antibody binding was detected by using species-specific (anti-goat) Amersham WB Cy3 secondary antibody (1:1000 in 3% BSA in TBS; Amersham, GE Healthcare) for 1h at RT. Following several washing steps in standard TBS-Tween20 (TBST) and finally in TBS, membranes were dried before scanning at auto excitation. Automated image analysis was performed with the Amersham WB evaluation software (v1.0, GE Healthcare, Little Chalfont, UK) with manual optimization if necessary. Target specific immunoreactivities were normalized to total protein intensities and statistically analysed.

#### Diet-induced obesity:

Control mice were fed a regular chow diet containing 57% of calories as carbohydrates, 34% of calories as protein, and 9% calories as a mixture of fatty acids. In contrast, high-fat diet (HFD) contained 35% of calories as carbohydrates, 20% calories as protein, and 45% calories as fatty acids (Research Diets). All experiments were performed in adult male mice during the period of P42-P170. Food intake and body weight were assessed weekly. Representative food intake shown was measured on the last week of HFD exposure.

#### Stereotaxic injection, viral particles:

Bilateral virus injections were made into the ARC of anesthetized 6/7-week-old male *Mfn2*^f/f^ mice placed into a stereotaxic apparatus (model 902; David Kopf instruments). AAV8-*Gfap-Gfp* (for control mice) or AAV8-*Gfap-Gfp-Cre* (Virus Vector Core, UNC; to generate astrocyte-specific knock-down) were applied at a volume of 300nl/hemisphere at coordinates (from bregma): anterior–posterior: −1.2 mm, lateral: ±0.3 mm, dorsal–ventral: −5.8 mm by using an air pressure system (injection time: 5 min). After surgery, mice were allowed to recover for 10 days before any dietary manipulation. Accurate virus injection into the ARC was verified by analyzing local GFP fluorescence. Mice with ‘missed’ or ‘partial’ hits were excluded. Specific AAV expression in ARC was also signified by double fluorescence labeling for GFP and GFAP.

#### Body composition:

Lean and fat mass were analyzed with EchoMRI (EchoMRI LLC)^95^.

#### Electron microscopy:

Mice (*n* ^3^ 4 per group) were anesthetized and transcardially perfused with freshly prepared 4% PFA and 0.1% glutaraldehyde as reported^95,161^. After post-fixation overnight, vibratome sections (50 μm) containing the ARC were immunostained with rabbit anti-POMC (1:7,500, H-029-30, Phoenix Pharmaceuticals,) or mouse anti-GFAP (1:4,500, Sigma) primary antibodies. After overnight incubation at 22–24 °C, sections were washed with 0.1M phosphate buffer (PB; pH7.4), incubated with either biotin-conjugated donkey anti-rabbit or donkey anti-mouse IgG secondary antibodies (1:250, Jackson ImmunoResearch) for 2h, washed again, placed in pre-formed avidin–biotin complex (Vector Laboratories), and developed with 3,3-diaminobenzidine and 0.01% H_2_O_2_. Sections were then osmicated (1% OsO_4_ for 15 min), and dehydrated in an ascending series of ethanol. During dehydration, 1% uranyl acetate was added to the 70% ethanol step to enhance ultrastructural membrane contrast. Flat embedding in Durcupan followed dehydration. Ultrathin sections were cut on a Leica ultra-microtome, collected on Formvar-coated single-slot grids, and analyzed with a Tecnai 12 Biotwin electron microscope (FEI) with an AMT XR-16 camera^161^,^162^.

#### Quantification of mitochondria, glial coverage, and synaptic input:

Hypothalamic sections containing either POMC^+^ or GFAP^+^ cells with a visible nucleus were analyzed by electron microscopy. Mitochondrial cross-sectional area was calculated. For glia coverage and synaptic inputs, a blinded investigator scored the percentage of glia and the number of synapses per POMC^+^ neuron in high-magnification images (>4.800x)^95,161^.

#### Statistics:

Experimental data were analyzed using GraphPad Prism 8.0.2 (GraphPad). Changes in *Fos* expression in the SCN (circadian activity and TRAP2 mice at CT6, CT18; n = 3 male mice per condition, P = 1) and PVN (TRAP mice after acute stress; *n* = 7 male mice per condition, P = 0,017) were analyzed across neurons and/or astrocytes (multiple groups, as applicable) with two-tailed *t*-test with Welch’s correction. Morphological parameters of astrocytes and synapses within ARC, as well as phenotypic (metabolic) parameters in the *Mfn2*^f/f^ mouse model under control condition and HFD-induced obesity were compared using two-tailed Student’s *t*-test; sample size was at least 4 animals per condition; experiments were performed in adult male mice at the age of 6–25 weeks. Data were expressed as means ± s.e.m. throughout, except in box-and-whisker plots that show medians ± s.e.m. Statistical significance was defined as *p < 0.05; **p < 0.01; ***p < 0.001. Statistical comparisons of classification models performance^146,147^, DABEST multiple groups comparisons of the magnitude and specificity rankings of ligand-receptor pairs^86–88^, and differential genes expression testing^120,123^ were described in corresponded sections above.

## Data Availability

All scRNA-seq datasets had been deposited in GEO previously, and re-analyzed here (for accession numbers and metadata see ED Table 1). Other experimental data will be made available upon reasonable request.
